# Supplementary material for: Unequivocal identification of two-bond heteronuclear correlations in natural products at nanomole scale by i-HMBC
Source: Nat Commun. 2023 Apr 3;14:1842. doi: 10.1038/s41467-023-37289-z (PMC10070429; doi:10.1038/s41467-023-37289-z)
Supplement: Supplementary file 1 — Supplementary Information [file 41467_2023_37289_MOESM1_ESM.pdf]

Supplementary Information

Unequivocal Identification of Two-Bond Heteronuclear Correlations in Natural Products at Nanomole Scale by i-HMBC

Yunyi Wang[1], Aili Fan[2], Ryan D. Cohen[1], Guilherme Dal Poggetto[1], Zheng Huang[3], Haifeng Yang[3], Gary E. Martin[4], Edward C. Sherer[1], Mikhail Reibarkh[1,*], Xiao Wang[1,*]

[1] Analytical Research & Development, Merck & Co., Inc., Rahway, NJ 07065, USA

[2] State Key Laboratory of Natural and Biomimetic Drugs, School of Pharmaceutical Sciences, Peking University, Beijing 100191, P.R. China

[3] Process Research & Development, Merck & Co., Inc., Rahway, NJ 07065, USA

[4] Department of Chemistry and Biochemistry, Seton Hall University, South Orange, NJ 07079, USA

[*] E-mail: xiao.wang1@merck.com, mikhail_reibarkh@merck.com

Table of Contents

Supplementary Methods

I. Synthesis and NMR Analysis of ^{13}C-Labeled Ethyl Acetate (EtOAc) 4

Supplementary Figure 1. ^{1}H spectrum of **1b** (600 MHz, $CDCl_3$, 298 K) 5

Supplementary Figure 2. ^{13}C{^{1}H} spectrum of **1b** (126 MHz, $CDCl_3$, 298 K) 6

Supplementary Figure 3. $^{2\text{-}3}\Delta^{1}$H($^{13/12}$C) measurements of EtOAc at natural isotope abundance with 4 s – 0.2 s AQ ($CDCl_3$, 800 MHz, 298 K). 7

Supplementary Figure 3. (continued) 8

II. Production, Isolation and NMR Studies of Homodimericin B 9

Supplementary Figure 4. ^{1}H spectrum of **11** (600 MHz, $CDCl_3$, 298 K) 10

Supplementary Figure 5. ^{13}C{^{1}H} spectrum of **11** (151 MHz, $CDCl_3$, 298 K) 11

Supplementary Figure 6. ^{1}H-^{1}H COSY spectrum of **11** (600 / 600 MHz, $CDCl_3$, 298 K) 12

Supplementary Figure 7. ^{1}H-^{13}C multiplicity-edited HSQC spectrum of **11** (600 / 151 MHz, $CDCl_3$, 298 K) 13

Supplementary Figure 8. ^{1}H-^{13}C i-HMBC spectrum of **11** (600 / 151 MHz, $CDCl_3$, 298 K) ... 14

Supplementary Figure 9. ^{1}H-^{1}H NOESY spectrum of **11** (600 / 600 MHz, $CDCl_3$, 298 K, mixing time = 0.5 s) ... 15

Supplementary Table 1. Measured isotope shift differences for **11**, in ppb. ... 16

Supplementary Figure 10. Selected i-HMBC slices of homodimericin B (**11**) showing chemical shifts and isotope shift measurements. (600 MHz, $CDCl_3$, 298 K) ... 17

Supplementary Figure 10. (continued) ... 18

III. NMR Studies of Calicheamicin γ_1^{I} ... 19

Supplementary Figure 11. ^{1}H spectrum of **12** (599 MHz, CD_3CN, 298 K) ... 20

Supplementary Figure 12. $^{13}C\{^{1}H\}$ spectrum of **12** (151 MHz, CD_3CN, 298 K) ... 21

Supplementary Figure 13. ^{1}H-^{1}H COSY spectrum of **12** (599 / 599 MHz, CD_3CN, 298 K) ... 22

Supplementary Figure 14. ^{1}H-^{13}C HSQC spectrum of **12** (599 / 151 MHz, CD_3CN, 298 K) ... 23

Supplementary Figure 15. ^{1}H-^{13}C i-HMBC spectrum of **12** (599 / 151 MHz, CD_3CN, 298 K) ... 24

Supplementary Figure 16. Key HMBC and i-HMBC correlations in **12**. ... 25

Supplementary Table 2. Measured isotope shift differences for **12**, in ppb. ... 26

Supplementary Figure 17. Selected i-HMBC slices of **12** showing chemical shifts and isotope shift measurements. (600 MHz, CD_3CN, 298 K) ... 27

Supplementary Figure 17. (continued) ... 28

IV. NMR Studies of Cryptospirolepine ... 29

Supplementary Figure 18. Structure revision of cryptospirolepine (**13**) ... 30

Supplementary Figure 19. i-HMBC analysis of **13b** ... 31

Supplementary Figure 20. ^{1}H spectrum of **13b** (600 MHz, CD_3OD, 298 K) ... 31

Supplementary Figure 21. ^{1}H-^{13}C i-HMBC spectrum of 1**3b** (600 / 151 MHz, CD_3OD, 298 K) ... 33

V. i-HMBC NMR Data and Spectra for Strychnine ... 34

Supplementary Table 3. Measured isotope shift differences for **4**, in ppb. ... 34

Supplementary Figure 22. Selected i-HMBC slices of **4** showing chemical shifts and isotope shift measurements. (600 MHz, $CDCl_3$, 298 K) ... 35

Supplementary Figure 22. (continued) ... 36

Supplementary Figure 22. (continued) ... 37

Supplementary Figure 22. (continued) ... 38

VI. i-HMBC NMR Data for Linalool, MTBE, Hydrochlorothiazide, Caffeine, Sulfamethoxazole, Prednisone, and Trimethoprim ... 39

Supplementary Table 4. Measured isotope shift differences for **2**, in ppb. ... 39

Supplementary Table 5. Measured isotope shift differences for **3**, in ppb. ... 39

Supplementary Table 6. Measured isotope shift differences for **5**, in ppb. 39
Supplementary Table 7. Measured isotope shift differences for **6**, in ppb. 40
Supplementary Table 8. Measured isotope shift differences for **7**, in ppb. 40
Supplementary Table 9. Measured isotope shift differences for **8**, in ppb. 41
Supplementary Table 10. Measured isotope shift differences for **9**, in ppb. 42
VII. i-HMBC and i-D-HMBC Pulse Sequences 43
Modified HMBC pulse sequence to improve line shape for i-HMBC... 43
Modified D-HMBC pulse sequence to improve line shape for D-i-HMBC with mild adiabatic decoupling 46
Supplementary Figure 23. D-i-HMBC pulse sequence 46
VIII. S/N comparison of HMBC, i-HMBC and 1,1-ADEQUATE Pulse Sequences.. 50
Supplementary Figure 24. H-C correlations of strychnine used for S/N comparison. 50
Supplementary Table 11. S/N comparison for HMBC, i-HMBC and 1,1-ADEQUATE experiments. ... 51
IX. Comparison of measurement accuracy of signals with different S/N using peak picking versus line fitting, and effect of NUS 52
Supplementary Table 12. Measurement accuracy of $^{2\text{-}3}\Delta^{1}H(^{13/12}C)$ in ethyl acetate via i-HMBC depending on spectral resolution and S/N. 53
X. Nomenclature of isotope shift 54
Supplementary References.. 54

Supplementary Methods

I. Synthesis and NMR Analysis of ^{13}C-Labeled Ethyl Acetate (EtOAc)

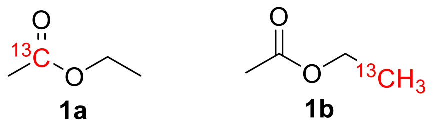

1a (99 atom% ^{13}C) was ordered from Sigma-Aldrich (catalog 279382-1G).

1b was synthesized from $^{13}CH_3CH_2OH$ (99 atom% ^{13}C), which was ordered from Sigma-Aldrich (catalog 427047-1G), following the procedure below.

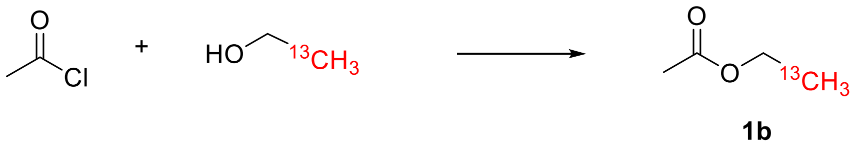

Ethyl alcohol-2-^{13}C (1 g, 21.2 mmol) was stirred and cooled in an ice bath. Acetyl chloride (1.4 mL, 1.54 g, 19.7 mmol) was added dropwise over 2 min. HCl gas was produced and collected by a drying tube with NaOH pellets. The reaction was further stirred for 20 min at room temperature before 5 mL of brine was added at 0 °C. The aqueous layer was discarded, and the organic layer was washed by saturated $NaHCO_3$ aq (5 mL X 3) and brine (5 mL). The organic layer was collected and dried over 4 Å molecular sieves overnight to afford 0.858 g ethyl-^{13}C acetate **1b** (45.3% yield).

^{1}H NMR (600 MHz, $CDCl_3$) δ 4.12 (qd, *J* = 7.1, 2.7 Hz, 2H), 2.04 (s, 3H), 1.25 (dt, *J* = 126.9, 7.1 Hz, 2H). ^{13}C NMR (126 MHz, $CDCl_3$) δ 171.04 (d, *J* = 2.5 Hz), 60.31 (d, *J* = 38.5 Hz), 14.14.

Supplementary Figure 1. ^{1}H spectrum of **1b** (600 MHz, $CDCl_3$, 298 K)

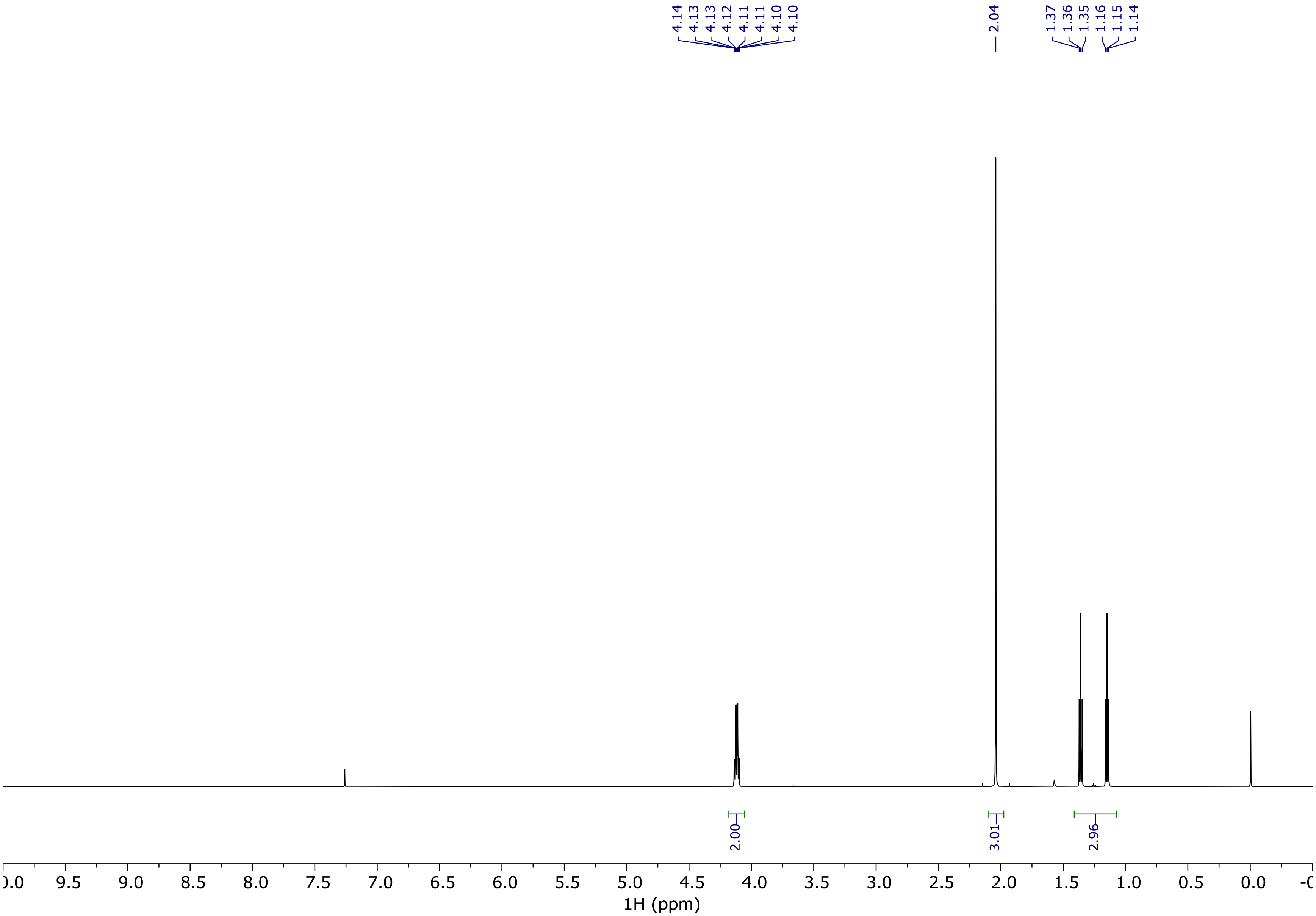

Supplementary Figure 2. $^{13}C\{^{1}H\}$ spectrum of **1b** (126 MHz, $CDCl_3$, 298 K)

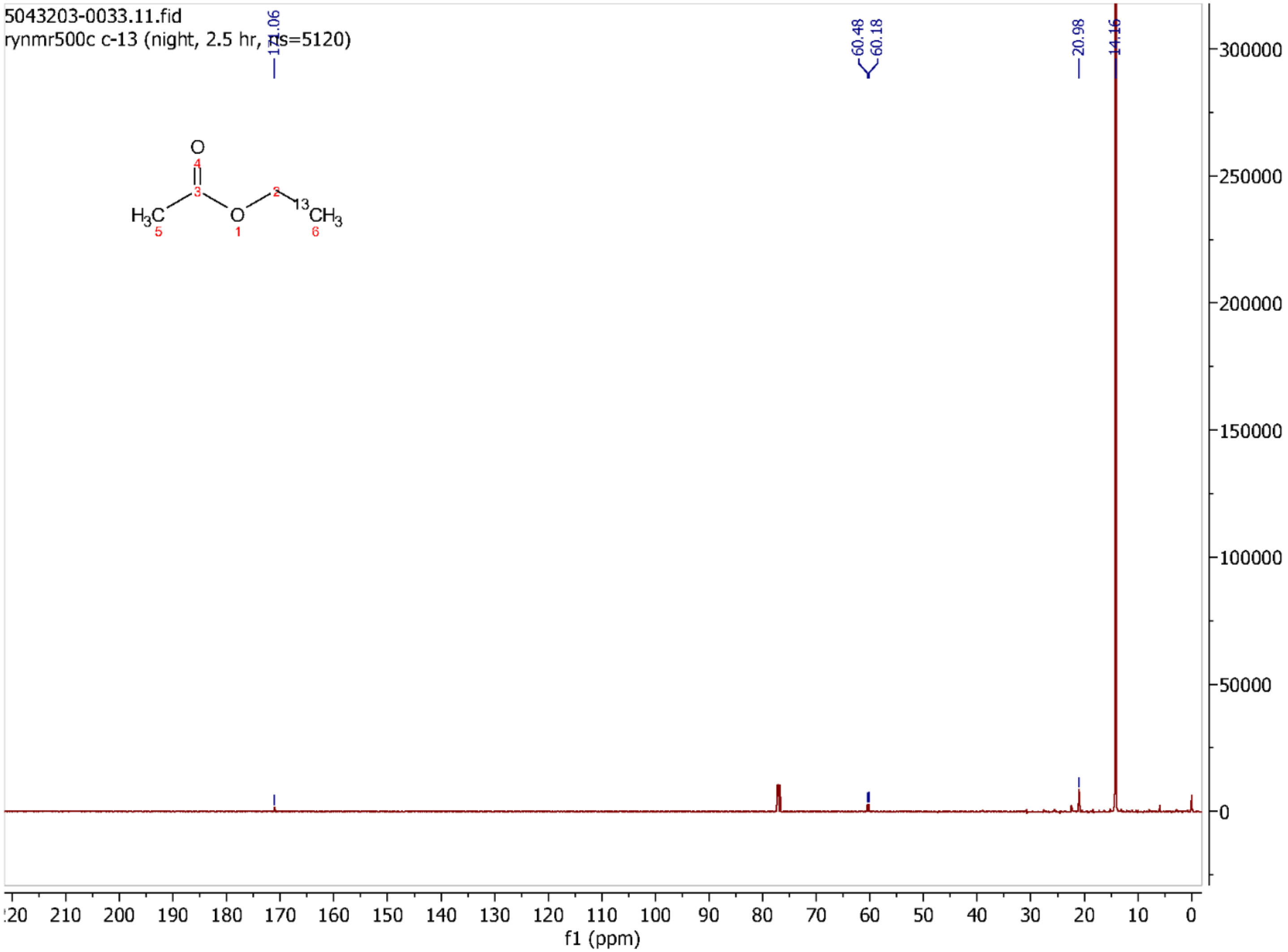

Supplementary Figure 3. $^{2\text{-}3}\Delta^{1}H(^{13/12}C)$ measurements of EtOAc at natural isotope abundance with 4 s – 0.2 s AQ ($CDCl_3$, 800 MHz, 298 K).

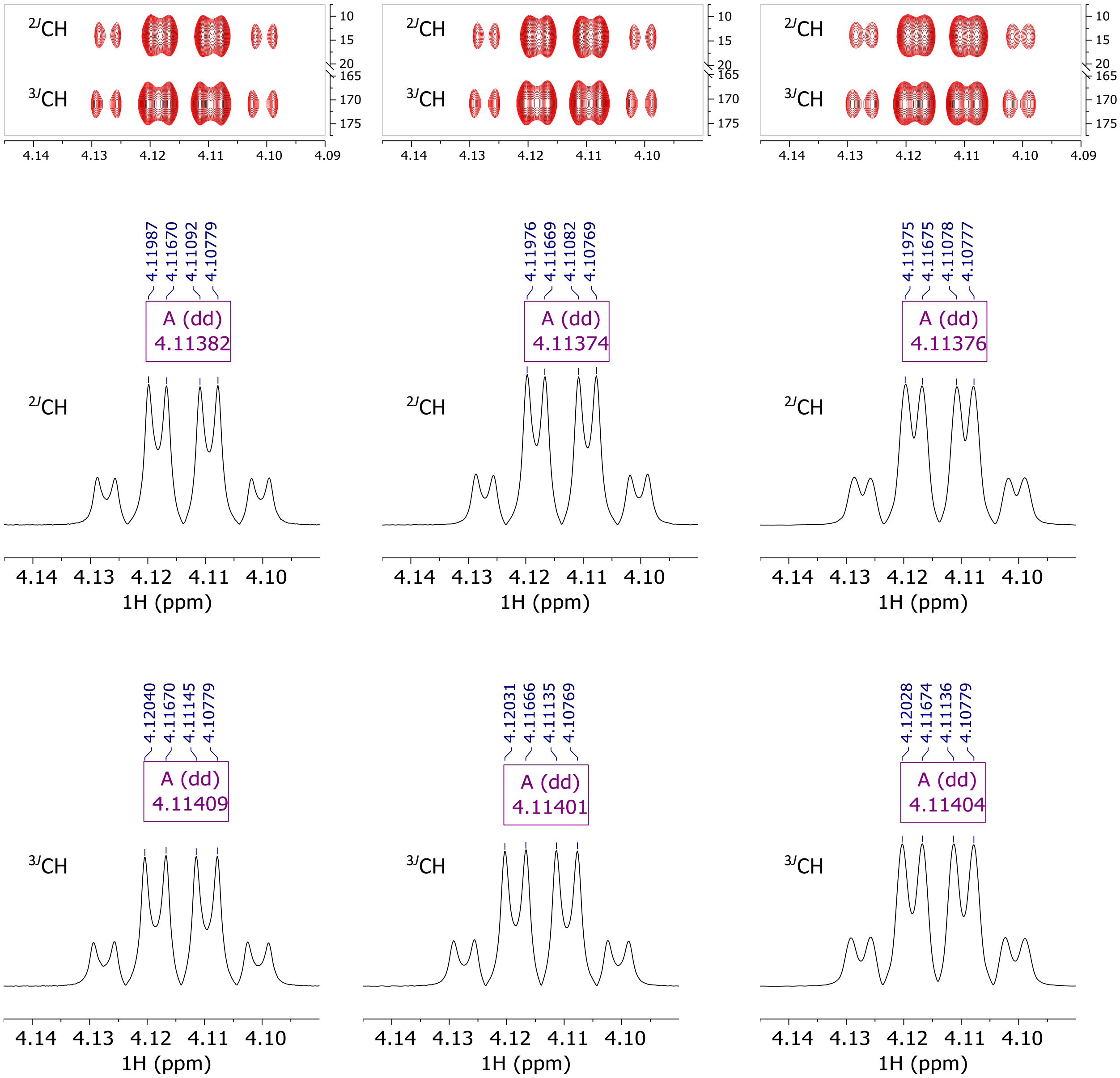

i-HMBC (AQ = 4 s) | i-HMBC (AQ = 2 s) | i-HMBC (AQ = 1 s)

$^{2\text{-}3}\Delta^{1}H(^{13/12}C)$ = -0.27 ppb | $^{2\text{-}3}\Delta^{1}H(^{13/12}C)$ = -0.27 ppb | $^{2\text{-}3}\Delta^{1}H(^{13/12}C)$ = -0.28 ppb

Supplementary Figure 3. (continued)

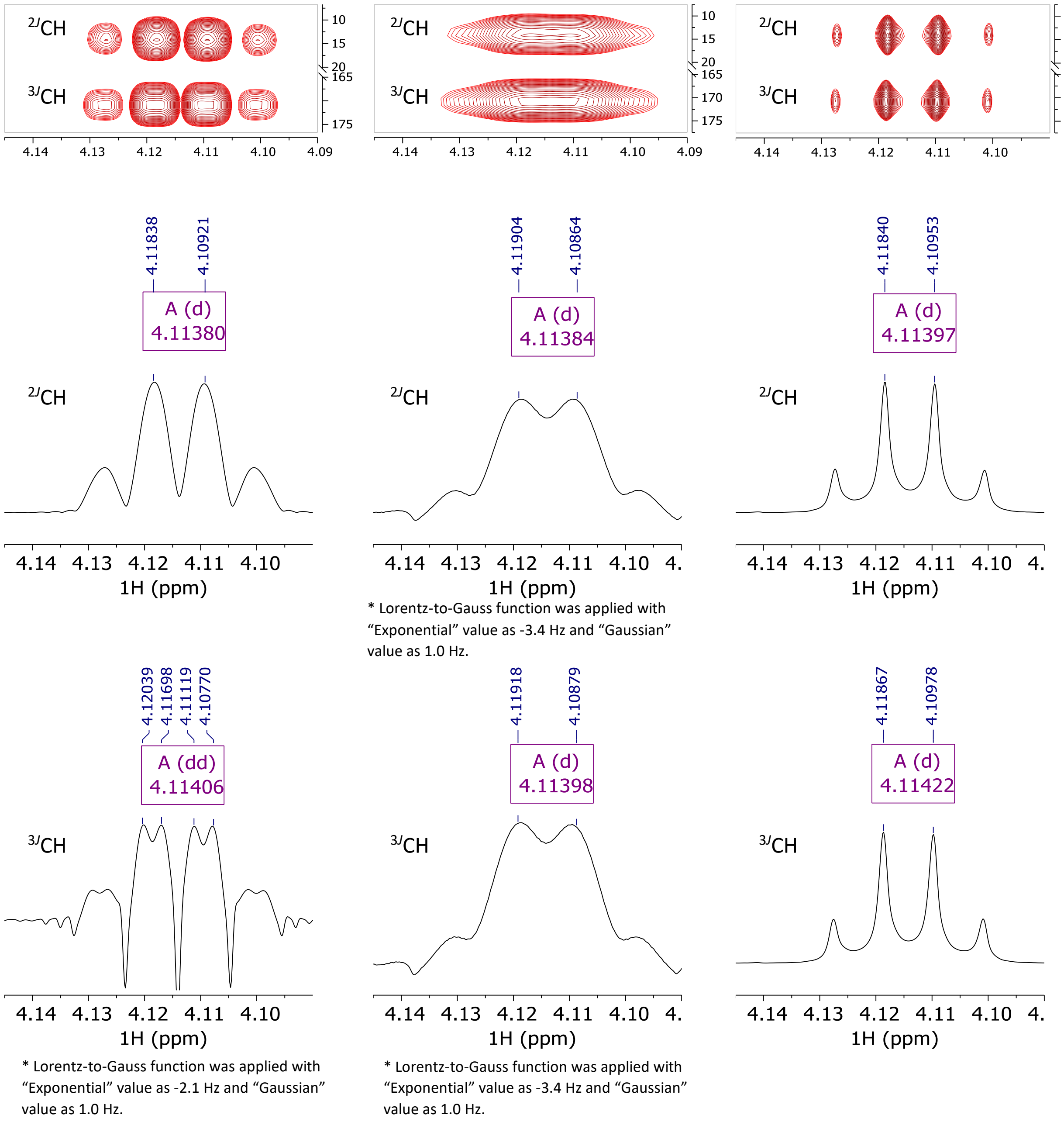

i-HMBC (AQ = 0.5 s)

$^{2\text{-}3}\Delta^{1}H(^{13/12}C)$ = -0.26 ppb

i-HMBC (AQ = 0.2 s)

$^{2\text{-}3}\Delta^{1}H(^{13/12}C)$ = -0.14 ppb

With 0.2 s AQ, the lineshape is too broad to accurately determine peak position.

D-i-HMBC (AQ = 2 s)

$^{2\text{-}3}\Delta^{1}H(^{13/12}C)$ = -0.25 ppb

II. Production, Isolation and NMR Studies of Homodimericin B

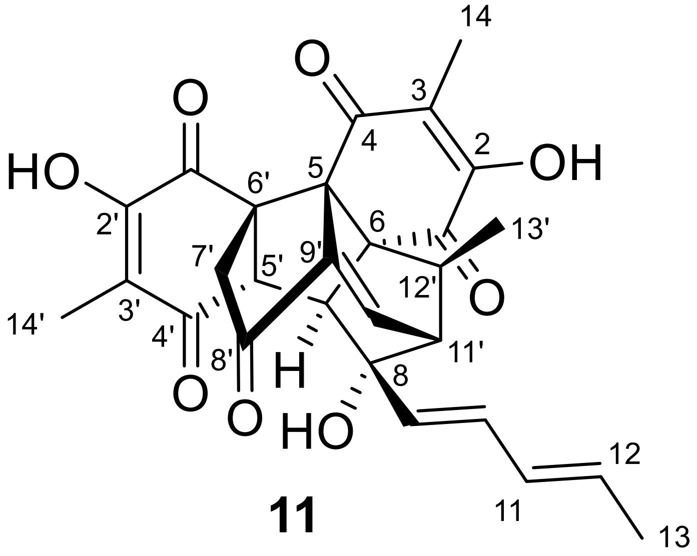

11

Fungal strain, fermentation, extraction and isolation of homodimericin B (8-*epi*-homodimericin A, **11**)

Trichoderma harzianum 3.9236 was purchased from China General Microbiological Culture Collection Center (CGMCC, Beijing, China). To obtain **11**, *T. harzianum* 3.9236 was cultivated at 28 °C for 7 days in PDB buffered to pH 8.0 using Na_2HPO_4/NaH_2PO_4. The cultures were extracted with EtOAc for 3 times. The EtOAc fraction was concentrated under reduced pressure and then subjected to ODS column eluting with a gradient of MeOH-H_2O (30:1 to 0:1) to afford homodimericin A (**10**) and homodimericin B (**11**).

Homodimericin B (**11**): ^{1}H NMR (600 MHz, $CDCl_3$) δ 6.60 (dd, *J* = 5.8, 1.1 Hz, 1H), 6.41 (dd, *J* = 15.7, 10.3 Hz, 1H), 6.15 (ddd, *J* = 15.2, 10.3, 1.4 Hz, 1H), 6.08 (d, *J* = 15.7 Hz, 1H), 5.86 (dq, *J* = 15.2, 6.8 Hz, 1H), 3.21 (dd, *J* = 5.6, 0.9 Hz, 1H), 3.02 (qdd, *J* = 7.1, 3.4, 1.1 Hz, 1H), 2.96–2.88 (m, 3H), 2.40 (d, *J* = 17.9 Hz, 1H), 2.05 (s, 3H), 1.94 (s, 3H), 1.80 (dd, *J* = 6.8, 1.4 Hz, 3H), 0.90 (d, *J* = 7.1 Hz, 3H); ^{13}C NMR (151 MHz, $CDCl_3$) δ 197.3, 194.9, 194.6, 194.2, 192.9, 154.6, 154.2, 140.0, 135.6, 133.3, 132.7, 130.5, 127.9, 127.1, 126.8, 88.7, 64.5, 64.4, 63.9, 62.6, 58.9, 52.6, 48.1, 42.0, 18.2, 12.2, 9.6, 9.3.

Supplementary Figure 4. ^{1}H spectrum of **11** (600 MHz, $CDCl_3$, 298 K)

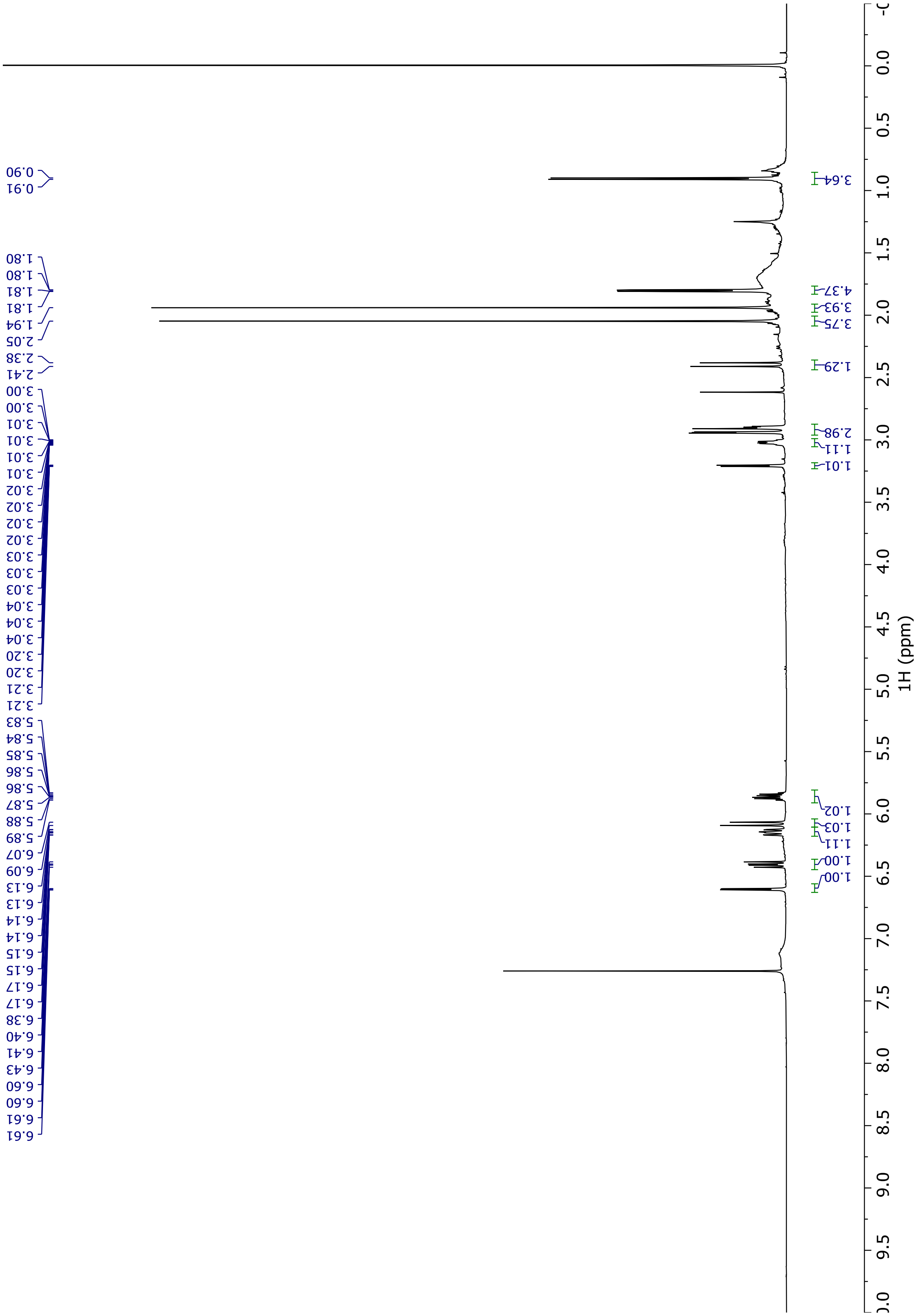

Supplementary Figure 5. $^{13}C\{^{1}H\}$ spectrum of **11** (151 MHz, $CDCl_3$, 298 K)

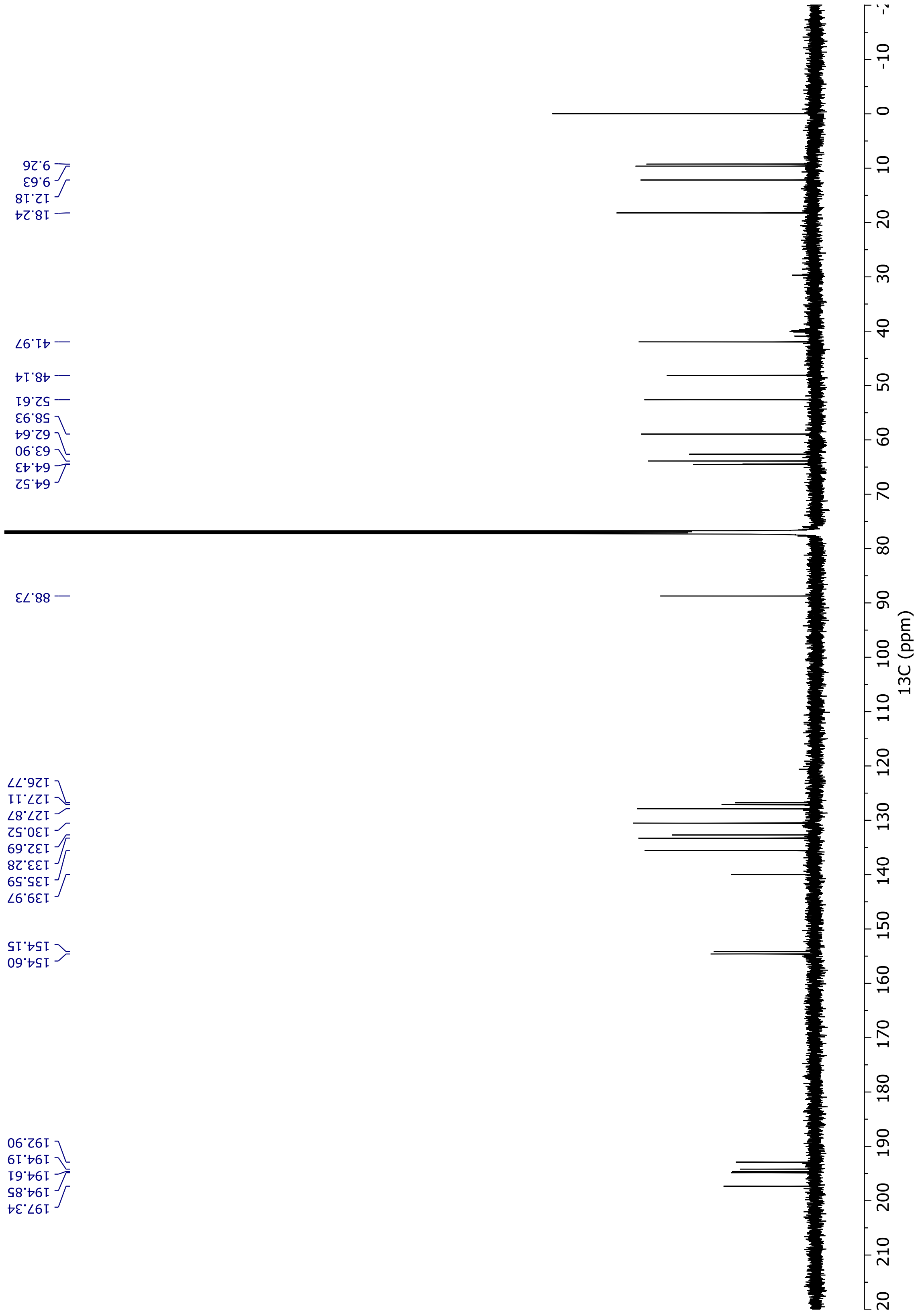

Supplementary Figure 6. ^{1}H-^{1}H COSY spectrum of **11** (600 / 600 MHz, $CDCl_3$, 298 K)

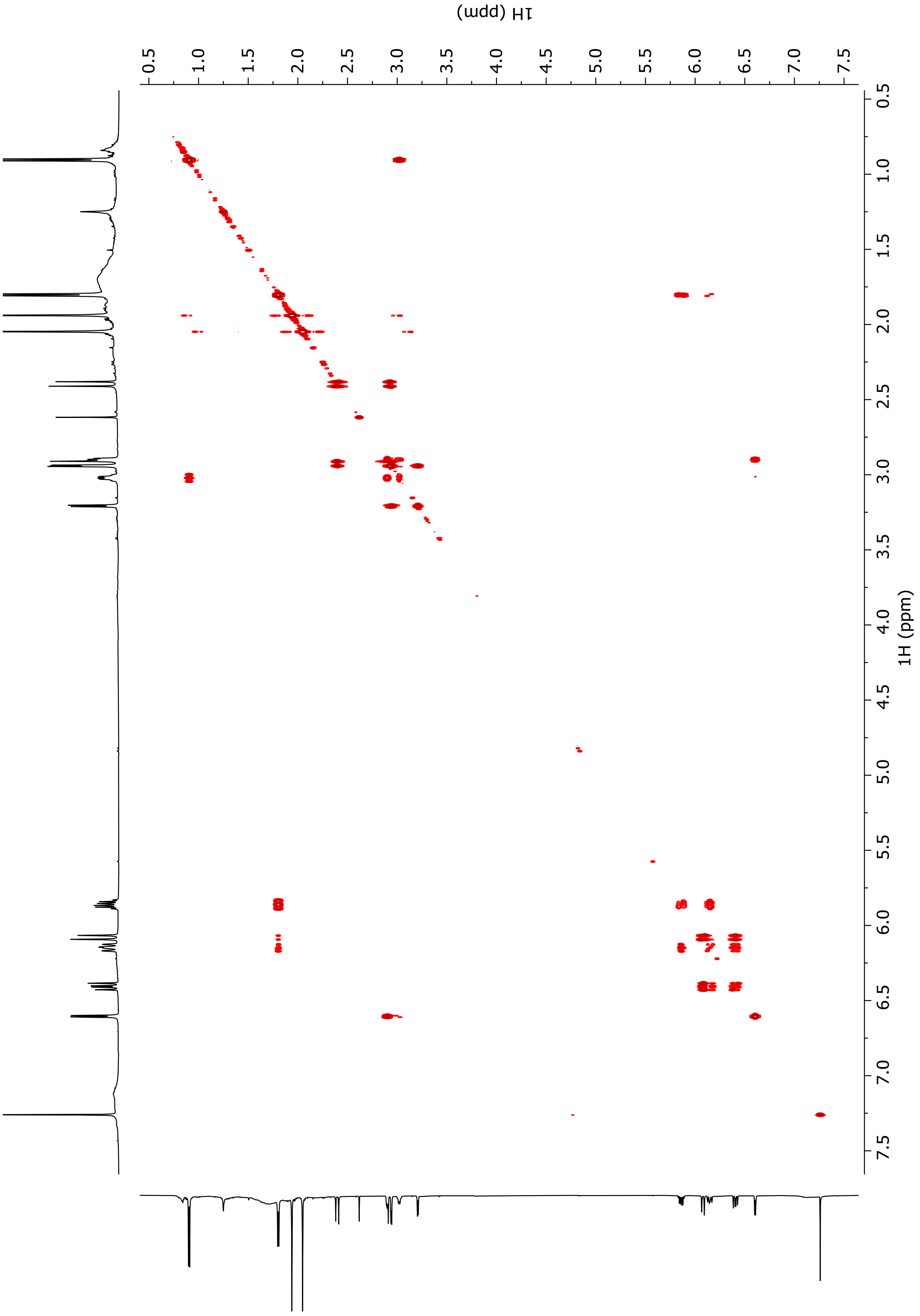

Supplementary Figure 7. ^{1}H-^{13}C multiplicity-edited HSQC spectrum of **11** (600 / 151 MHz, $CDCl_3$, 298 K)

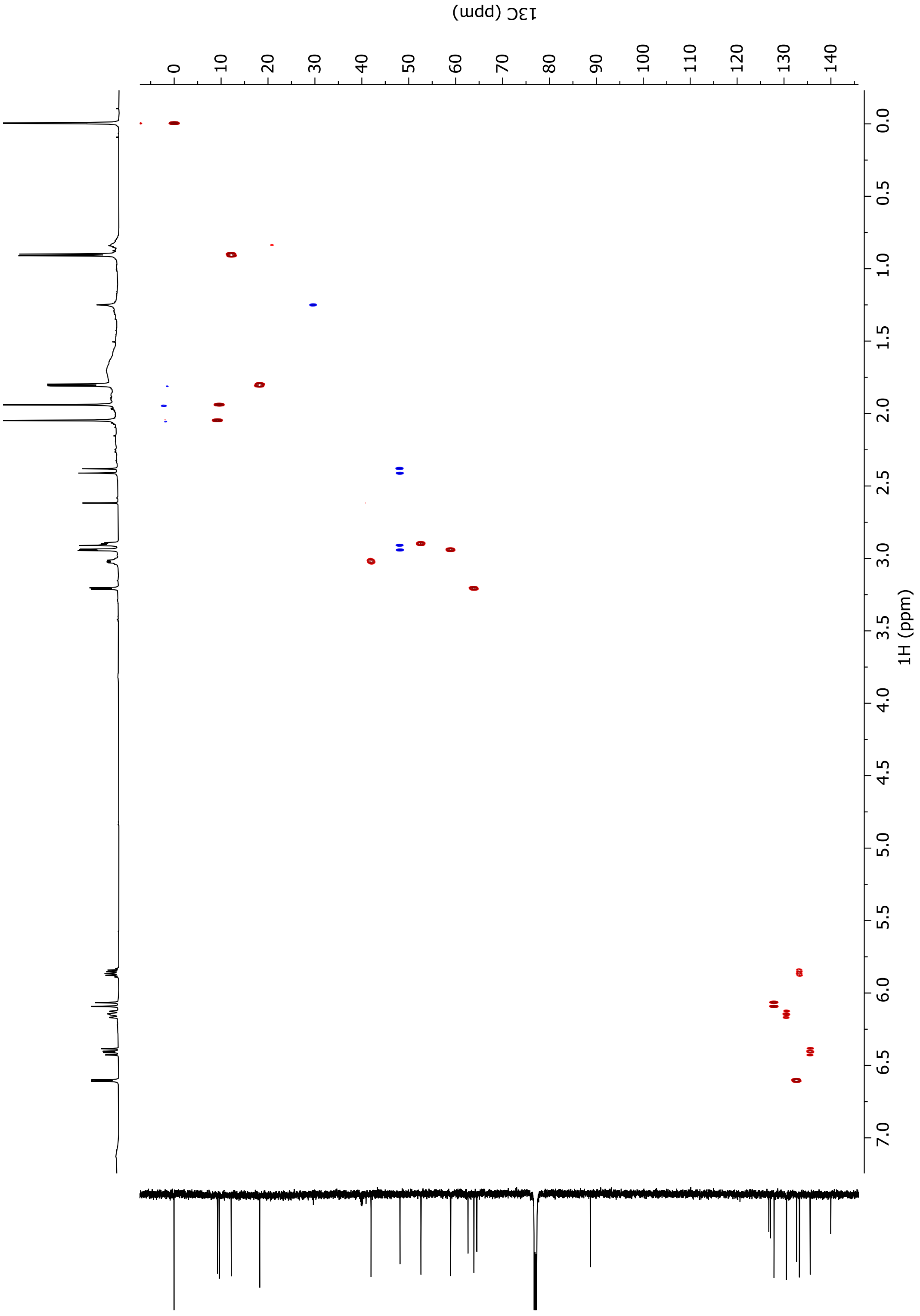

Supplementary Figure 8. ^{1}H-^{13}C i-HMBC spectrum of **11** (600 / 151 MHz, CDCl$_3$, 298 K)

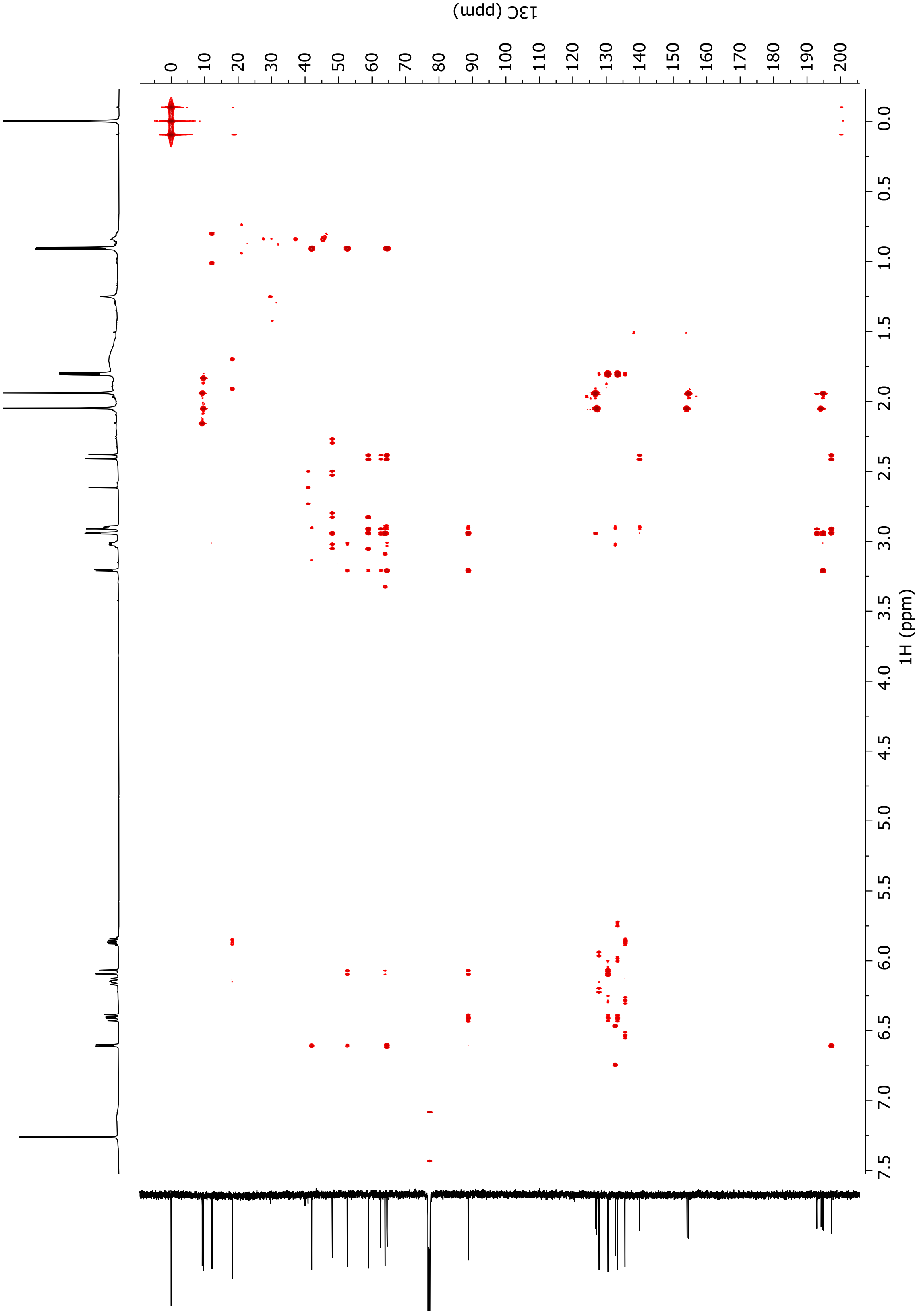

Supplementary Figure 9. ^1H-^1H NOESY spectrum of **11** (600 / 600 MHz, $CDCl_3$, 298 K, mixing time = 0.5 s)

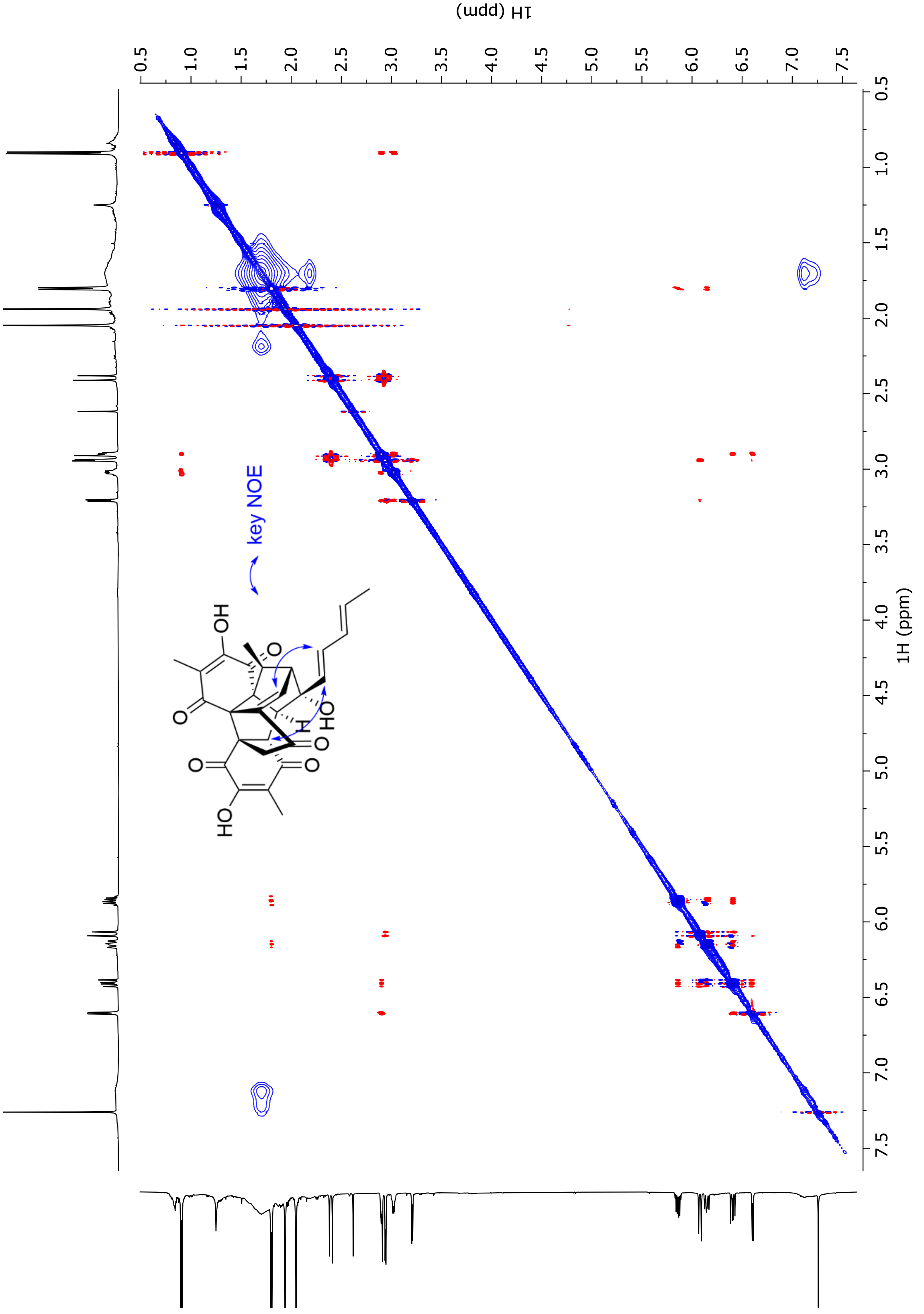

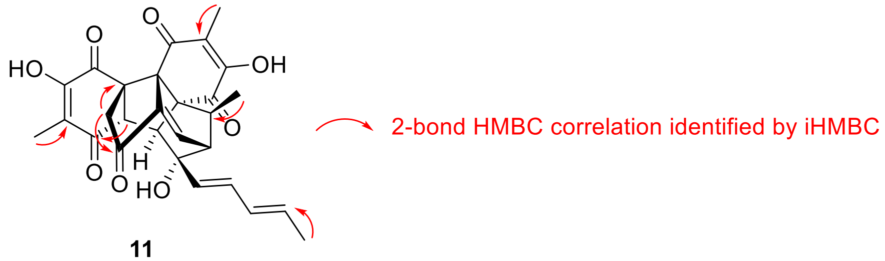

11

Supplementary Table 1. Measured isotope shift differences for **11**, in ppb.

	H13'	H13	H14'	H14	H7'	H5'
C12'	-0.45					
C7'						-0.08
C11'	0					
C5'					-0.11	
C6'					-0.57	
C5					-0.21	
C6	-0.03					
C8						0
C3'			-0.37			
C3				-0.42		
C11		0				
C12		-0.37				
C9'					0	
C2				-0.10		
C2'			-0.10			
C4				0		
C4'			0			-0.30
C8'					-0.49	

Supplementary Figure 10. Selected i-HMBC slices of homodimericin B (**11**) showing chemical shifts and isotope shift measurements. (600 MHz, $CDCl_3$, 298 K)

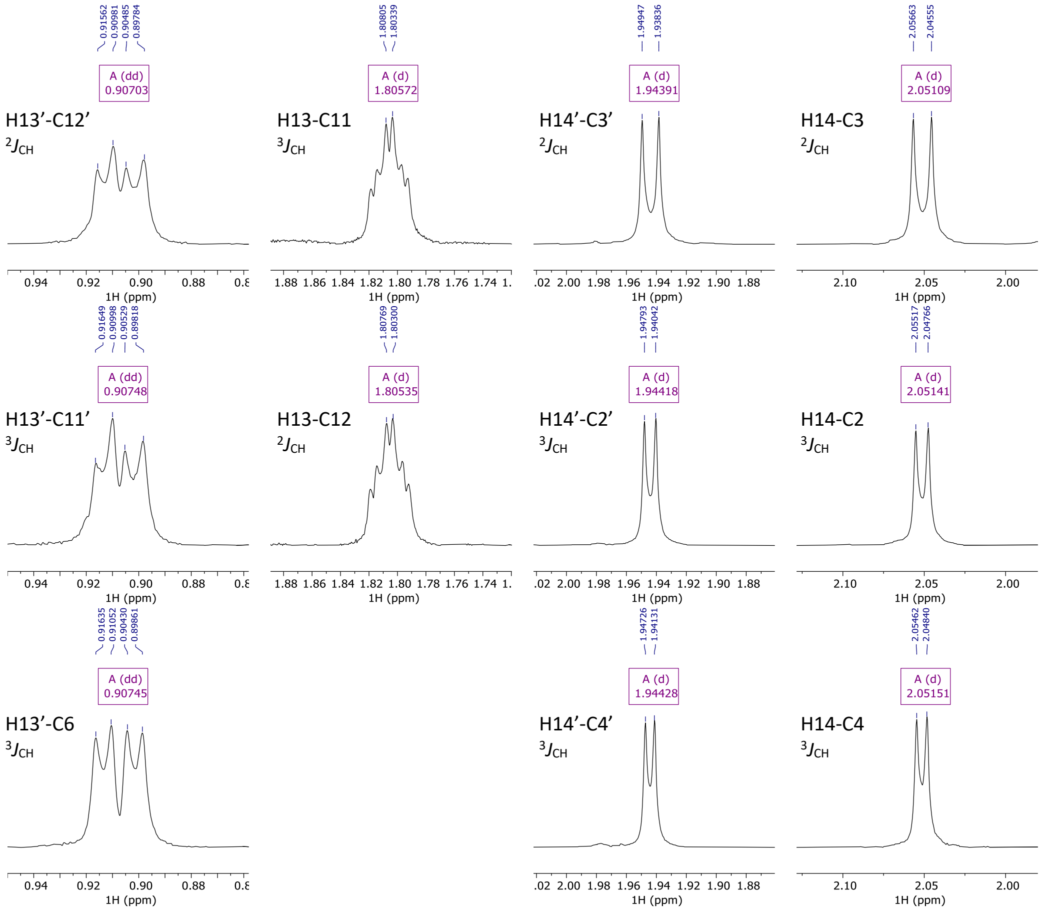

Supplementary Figure 10. (continued)

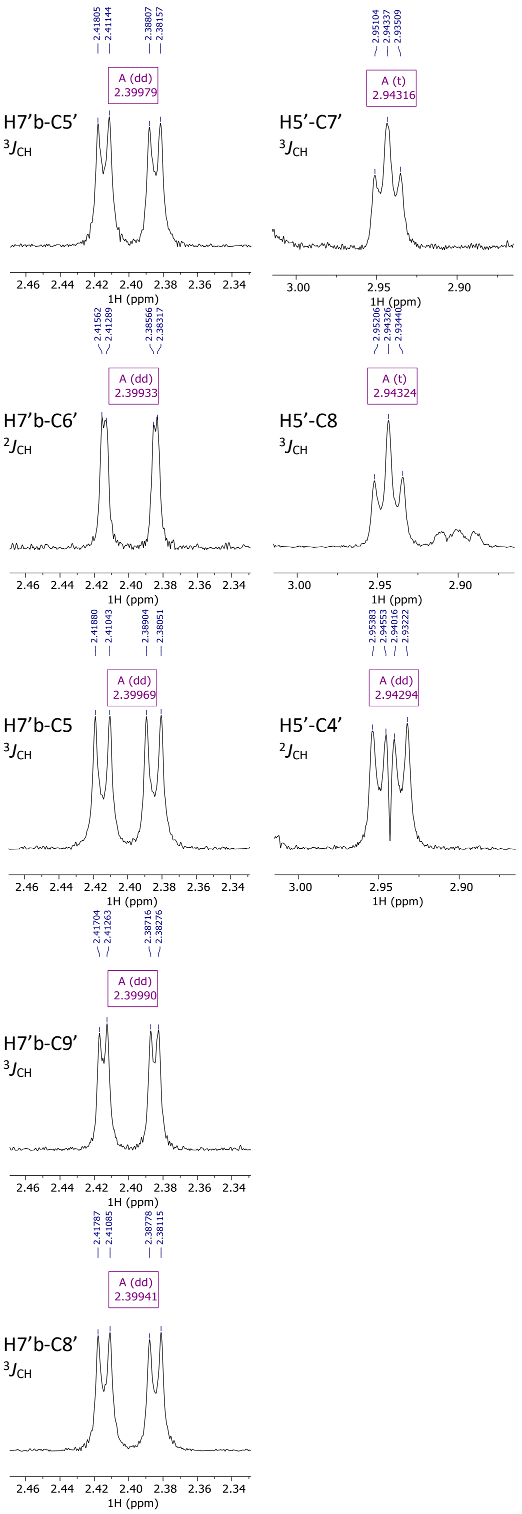

III. NMR Studies of Calicheamicin γ_1^I

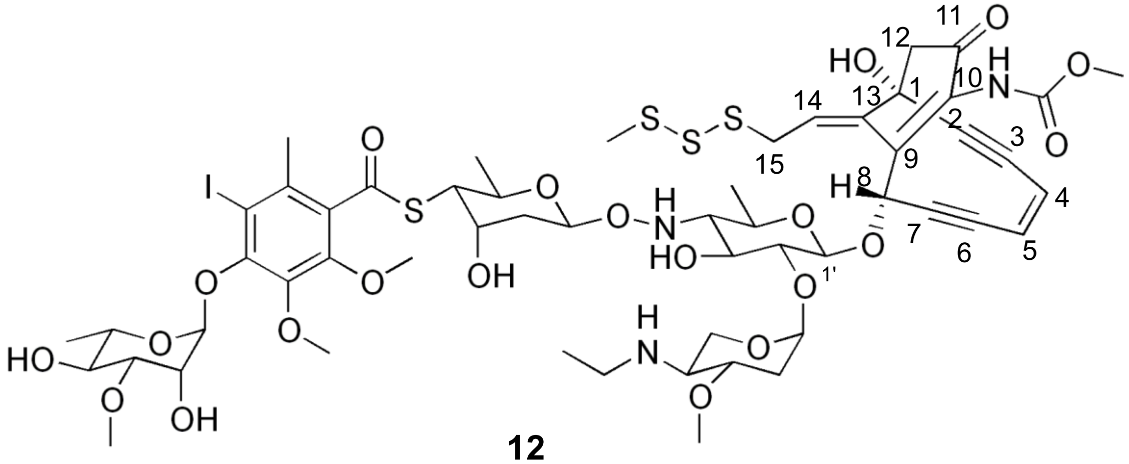

12

Calicheamicin γ_1^I (**12**): ^{1}H NMR (599 MHz, CD_3CN) δ 6.65 (d, *J* = 2.3 Hz, 1H), 6.34 (dd, *J* = 10.1, 5.1 Hz, 1H), 6.25 (d, *J* = 1.7 Hz, 1H), 6.07 (d, *J* = 9.5 Hz, 1H), 5.95 (dd, *J* = 9.5, 1.6 Hz, 1H), 5.66 – 5.62 (m, 1H), 5.55 (d, *J* = 1.8 Hz, 1H), 4.96 (dd, *J* = 10.2, 2.1 Hz, 1H), 4.92 (br, 1H), 4.60 (d, *J* = 7.7 Hz, 1H), 4.40 (d, *J* = 2.8 Hz, 1H), 4.20 (t, *J* = 3.1 Hz, 1H), 4.08 (dq, *J* = 9.8, 6.4 Hz, 1H), 4.04 – 3.91 (m, 3H), 3.88 (m, 4H), 3.81 (s, 3H), 3.80 – 3.76 (m, 1H), 3.75 – 3.64 (m, 4H), 3.60 – 3.53 (m, 4H), 3.51 – 3.44 (m, 7H), 3.37 (s, 3H), 3.24 – 3.18 (m, 2H), 3.02 (d, *J* = 17.6 Hz, 1H), 2.64 (d, *J* = 17.6 Hz, 1H), 2.63 – 2.55 (m, 1H), 2.53 (s, 3H), 2.52 – 2.42 (m, 2H), 2.34 (s, 3H), 2.32 – 2.22 (m, 2H), 1.91 (ddd, *J* = 13.4, 3.3, 2.1 Hz, 1H), 1.72 (ddd, *J* = 13.1, 10.2, 2.7 Hz, 1H), 1.47 (ddd, *J* = 12.7, 10.9, 3.6 Hz, 1H), 1.34 (d, *J* = 6.3 Hz, 3H), 1.29 (d, *J* = 6.2 Hz, 3H), 1.18 (d, *J* = 6.2 Hz, 3H), 1.06 (t, *J* = 7.1 Hz, 3H); ^{13}C NMR (151 MHz, CD_3CN) δ 193.5, 193.2, 156.7, 152.9, 151.6, 148.0, 144.2, 138.4, 134.2, 133.9, 131.4, 127.7, 126.3, 124.1, 104.7, 102.2, 101.2, 100.9, 98.7, 98.4, 94.2, 89.4, 83.8, 81.5, 78.9, 77.0, 72.7, 72.3, 72.0, 71.7, 71.7, 70.8, 70.7, 69.0, 68.5, 67.7, 63.1, 62.4, 61.6, 59.9, 57.4, 56.6, 54.2, 52.7, 52.0, 42.7, 40.5, 38.4, 34.6, 25.7, 23.2, 19.4, 18.4, 18.0, 14.9.

5 mg **12** was dissolved in 550 µL CD_3CN and used for NMR analysis.

i-HMBC parameters: for ^{1}H dimension (at 600 MHz), 13.6 ppm spectrum width (SW), 16384 points or 8192 complex points, 1 s AQ, resulted in 1 Hz FIDRES; for 13C dimension, 200 ppm SW, 512 F1 increments; 64 number of scans (NS); 2 s relaxation delay (D1); 50% NUS applied. Total experimental time was 14 h. During processing, a 90° sine square apodization was applied in the F1 dimension, and a 45° sine bell was applied in F2; 8x zero-filling was used to increase the number of points in the ^{1}H dimension to 128k (64k complex points). The exact i-HMBC peak positions (in ppm, with 5 decimals) were obtained by extracting the horizontal slices followed by peak picking in MestreLab's MNova.

Supplementary Figure 11. ^{1}H spectrum of **12** (599 MHz, CD_3CN, 298 K)

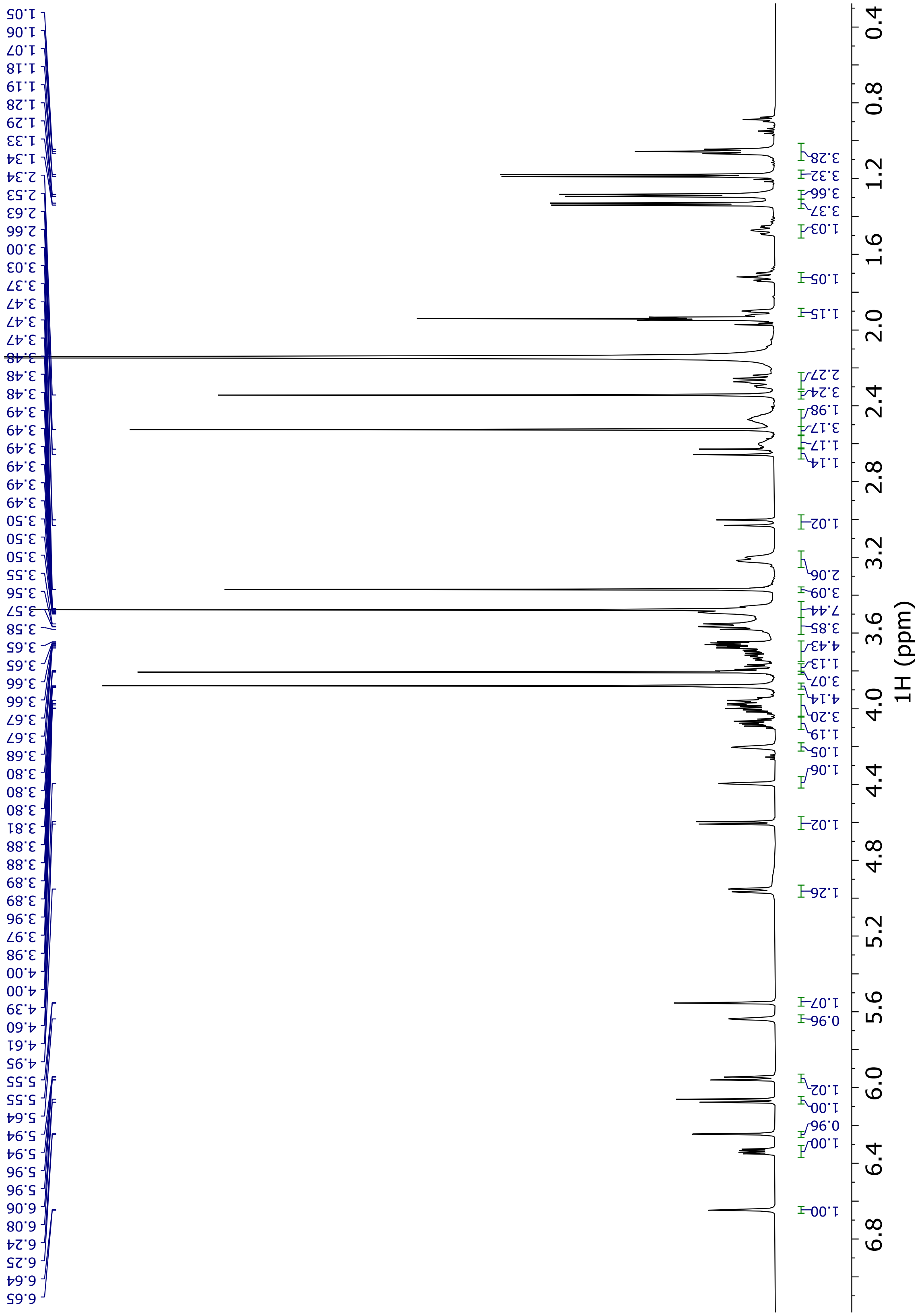

Supplementary Figure 12. $^{13}C\{^{1}H\}$ spectrum of **12** (151 MHz, CD_3CN, 298 K)

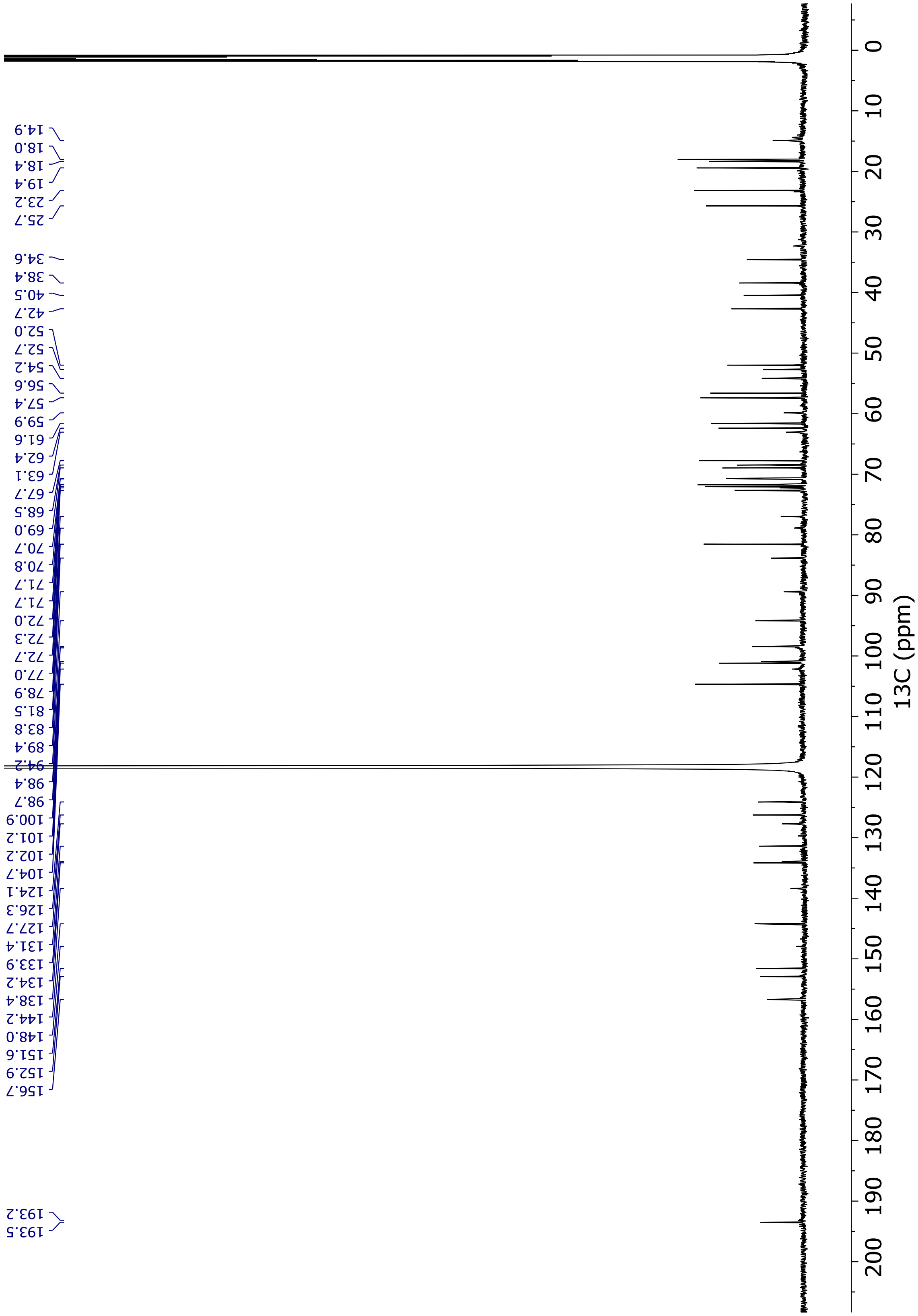

Supplementary Figure 13. ^1H-^1H COSY spectrum of **12** (599 / 599 MHz, CD_3CN, 298 K)

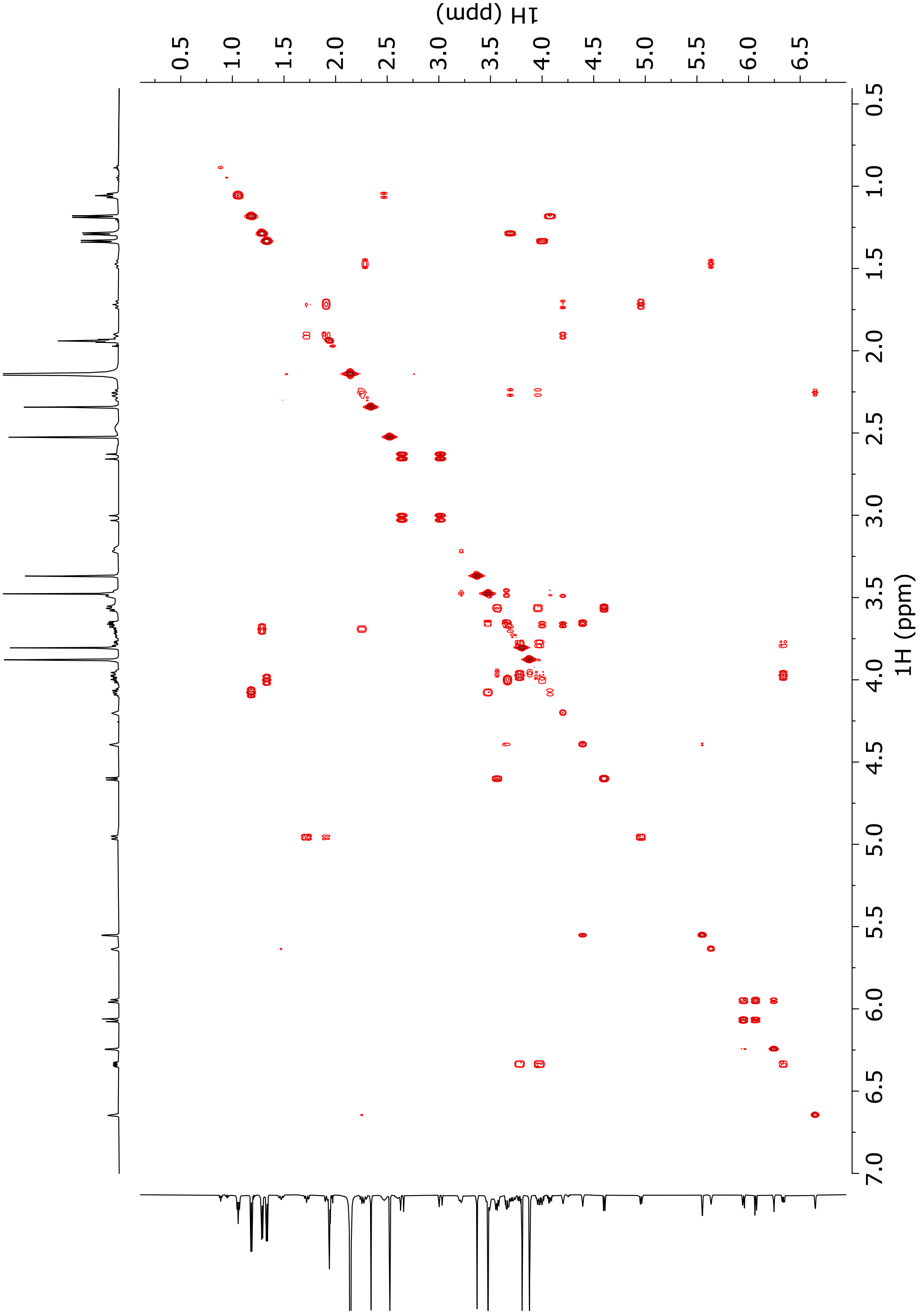

Supplementary Figure 14. ^{1}H-^{13}C HSQC spectrum of **12** (599 / 151 MHz, CD_3CN, 298 K)

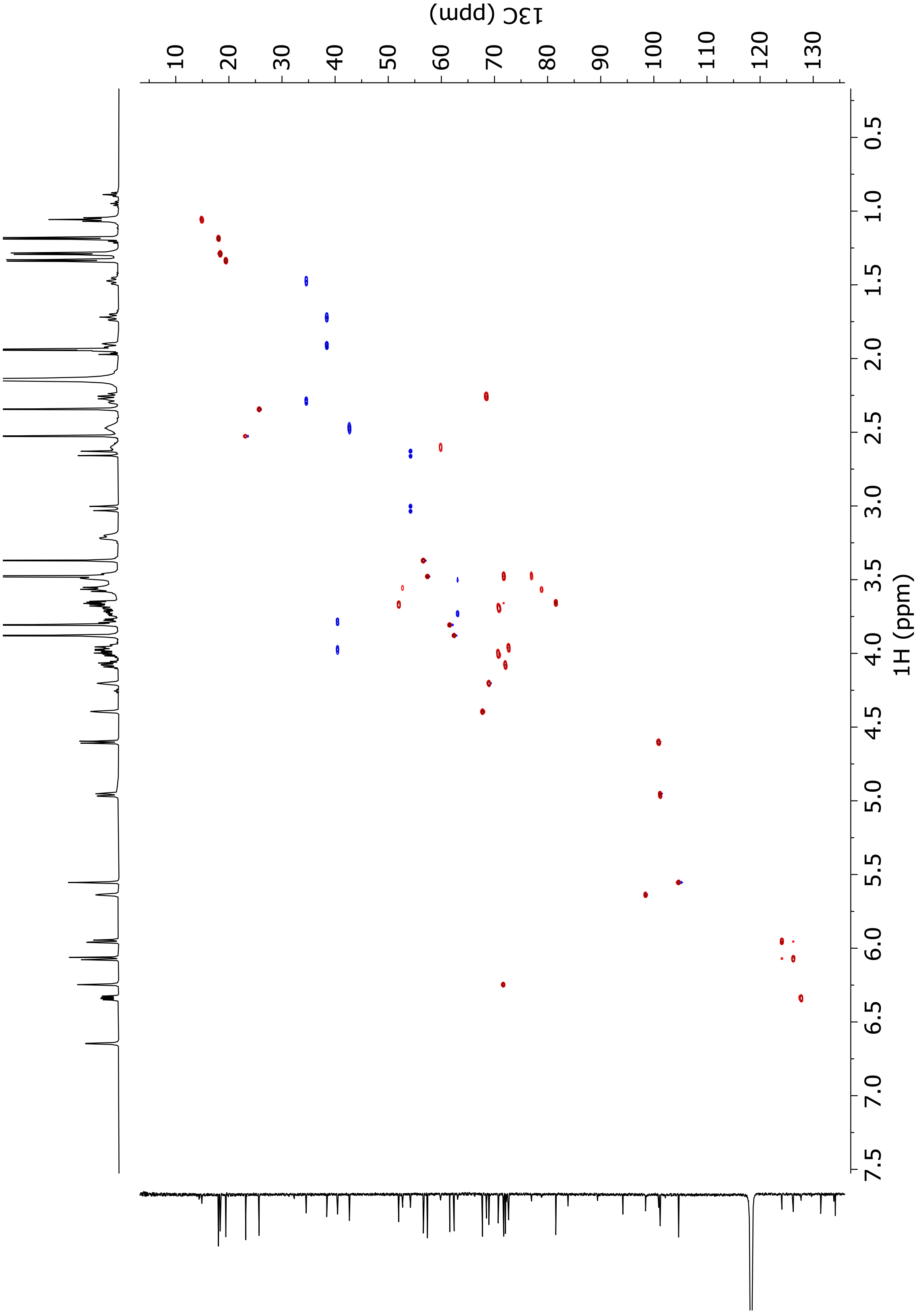

Supplementary Figure 15. ^{1}H-^{13}C i-HMBC spectrum of **12** (599 / 151 MHz, CD_3CN, 298 K)

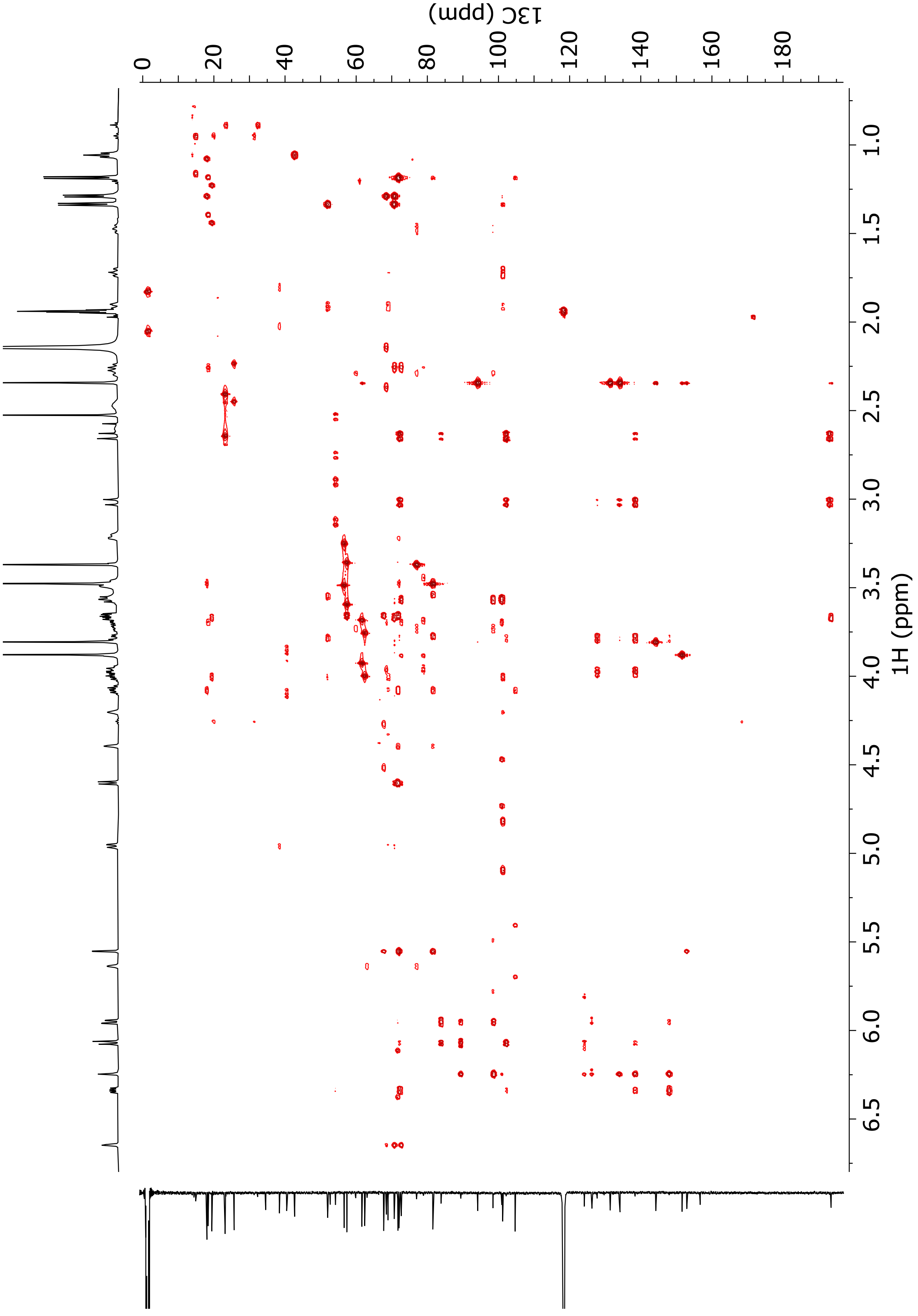

Supplementary Figure 16. Key HMBC and i-HMBC correlations in **12**.

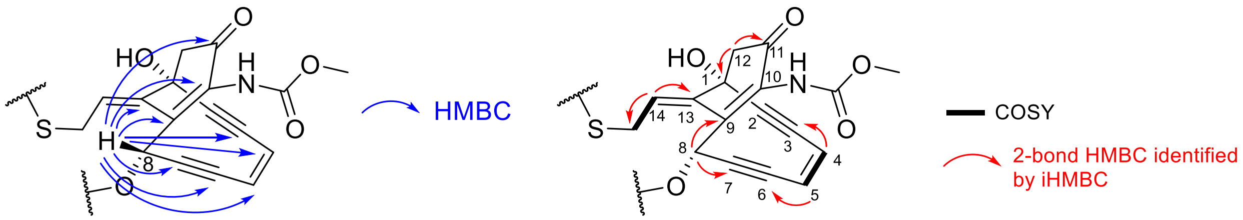

The original structure elucidation of the core structure of **12** in 1989 was extremely challenging. This was achieved through a combination of hydrolysis to partially remove saccharides followed by Bergman-type cyclization to convert the enediyne to a phenyl group. Extensive NMR, MS and single crystal X-ray diffraction analyses were then performed on the aromatized degradants.

The major difficulty during NMR structure elucidation of **12**, besides highly overlapped signals, was that too many long range HMBC correlations were observed, due to the high rigidity and conjugation of the core skeleton, which is not unusual for proton-deficient molecules. This significantly complicated data interpretation because a large number of reasonable test structures could be proposed. In the case of **12**, H8 showed nine HMBC correlations of moderate to strong intensity, ranging from 2- to 6-bonds, with C7, C6, C5, C4, C3, C9, C10, C11 and C13, as shown in Scheme S1 above.

When the i-HMBC methodology was employed, eight 2-bond HMBC correlations could be unambiguously identified, allowing straightforward and confident structure elucidation of the proton-deficient enediyne core of **12**.

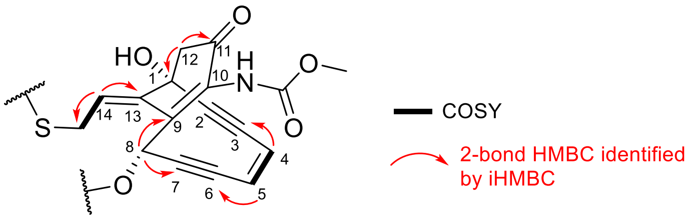

Supplementary Table 2. Measured isotope shift differences for **12**, in ppb.

	H14	H8	H4	H5	H12a	H12b
C15	-0.64					
C12	0					
C1	-0.10		0		-0.61	-0.82
C3			-0.37	-0.13		0
C6		0	-0.05	-0.34		
C7		-0.42		-0.15		
C1'		-0.04				
C2	-0.08		-0.02		-0.09	-0.21
C5		-0.13				
C4		-0.07				
C14						
C10		-0.17			0	
C13	-1.04	-0.04			0.00	-0.22
C9	-0.10	-0.30		0		
C11					-0.51	-0.51

Supplementary Figure 17. Selected i-HMBC slices of **12** showing chemical shifts and isotope shift measurements. (600 MHz, CD_3CN, 298 K)

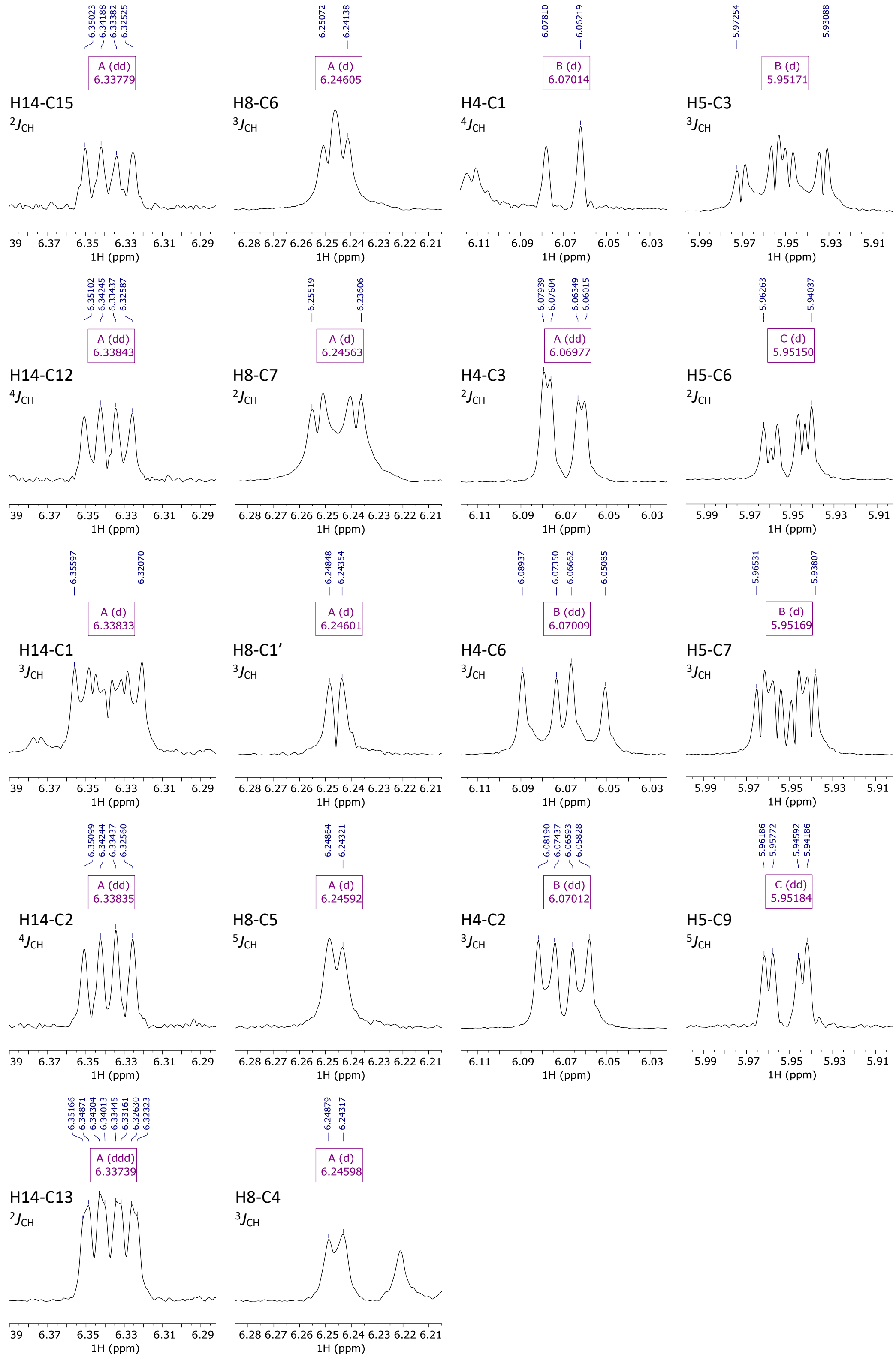

Supplementary Figure 17. (continued)

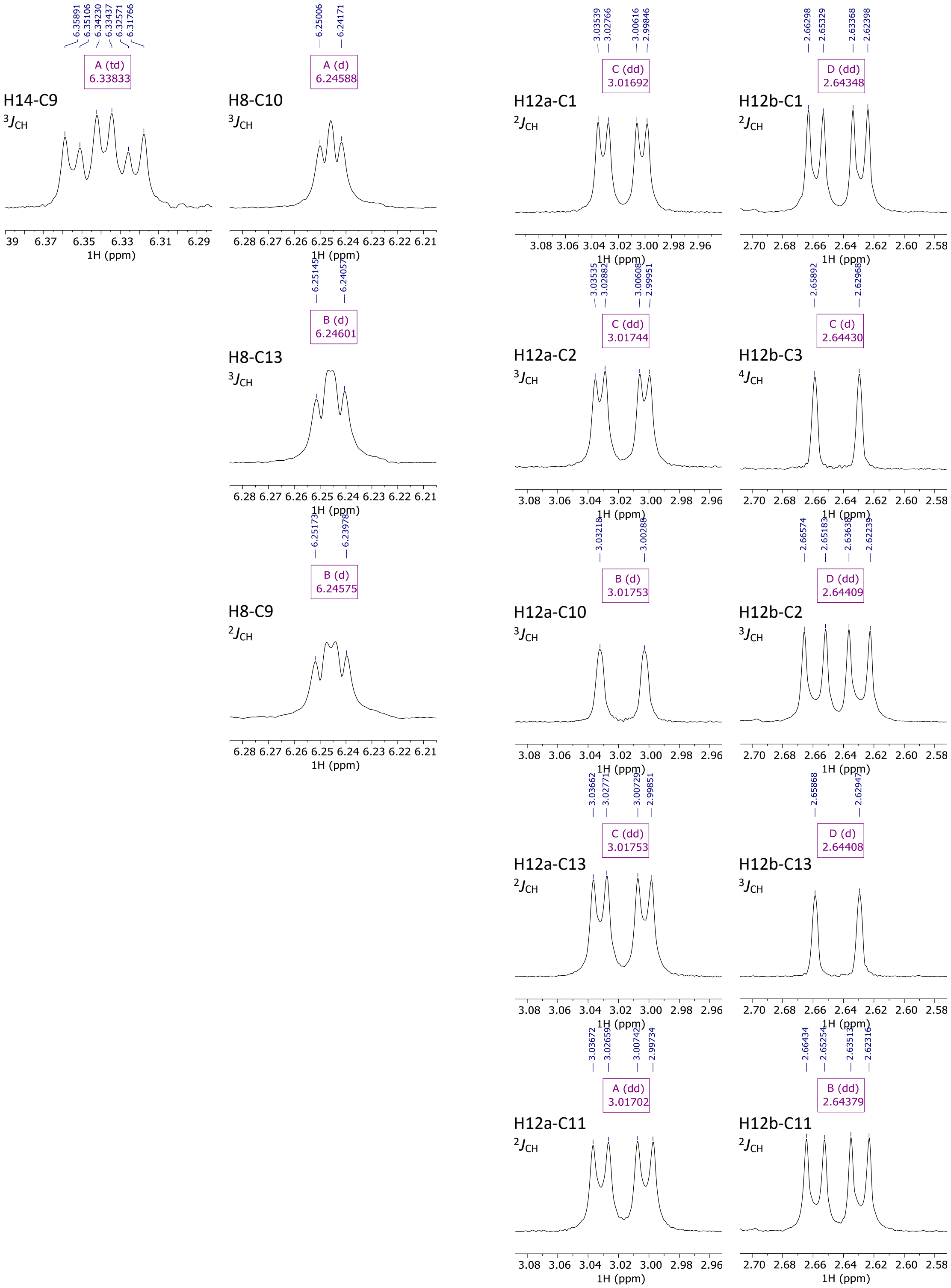

IV. NMR Studies of Cryptospirolepine

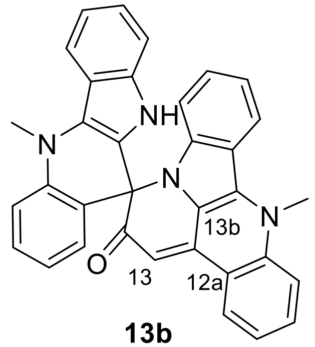

13b

Cryptospirolepine (**13b**): ^{1}H NMR (600 MHz, MeOD) δ 8.42 (d, *J* = 8.3 Hz, 1H), 8.31 (d, *J* = 8.4 Hz, 1H), 8.05 (d, *J* = 8.8 Hz, 2H), 7.89 (t, *J* = 7.8 Hz, 1H), 7.56 (t, *J* = 7.6 Hz, 1H), 7.30 – 7.20 (m, 2H), 7.15 – 7.08 (m, 2H), 7.08 – 7.00 (m, 3H), 6.96 – 6.93 (m, 1H), 6.75 (d, *J* = 8.5 Hz, 1H), 6.65 (ddd, *J* = 8.0, 6.0, 2.0 Hz, 1H), 4.51 (s, 3H), 4.06 (s, 3H). ^{13}C NMR was not acquired due to low amount of the sample.

0.14 mg **13b** was dissolved in 550 µL CD_3OD and used for NMR analysis.

i-HMBC parameters: for ^{1}H dimension (at 600 MHz), 13 ppm spectrum width (SW), 15624 points or 7812 complex points, 1 s AQ, resulted in 1 Hz FIDRES; for ^{13}C dimension, 90 ppm SW, 256 F1 increments; 512 number of scans (NS); 0.5 s relaxation delay (D1). Total experimental time was 57.3 h. During processing, a 90° sine square apodization was applied in the F1 dimension, and a 45° sine bell was applied in F2; 8x zero-filling was used to increase the number of points in the ^{1}H dimension to 128k (64k complex points). The exact i-HMBC peak positions (in ppm, with 5 decimals) were obtained by extracting the horizontal slices followed by line fitting in MestreLab's MNova.

Supplementary Figure 18. Structure revision of cryptospirolepine (13)

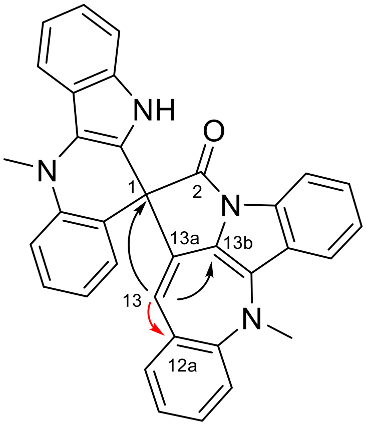

13a
original (1993)

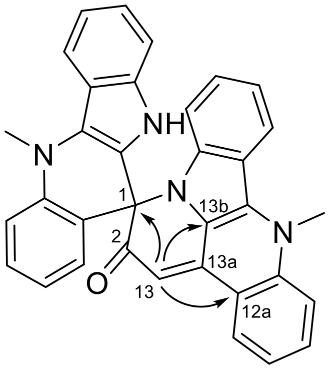

13b
revised (2015)

The misassignment of cryptospirolepine to **13a** was mainly because the HMBC correlation between H13 and C2 was not observed in the HMBC experiment optimized for 10 Hz (this ${}^{2}J_{CH}$ was calculated to be 0.03 Hz by DFT methods). In addition, the H13-H12a HMBC signal was assumed to be a 2-bond correlation. During the structure revision in 2015, it was found that the ${}^{2}J_{CH}$ between H13 and C2 was calculated by DFT to be 0.03 Hz which explained the missing HMBC correlation. In this case, i-HMBC could not observe the 2-bond correlation of H13-C2 for the same reason. However, i-HMBC could still be used to disprove the structure of **13a** assigned in 1993, since H13-C12a was clearly not a 2-bond correlation because it showed only a 0.02 ppb isotope shift difference from the 3-bond correlation H13-C13b, as illustrated below.

Supplementary Figure 19. i-HMBC analysis of **13b**

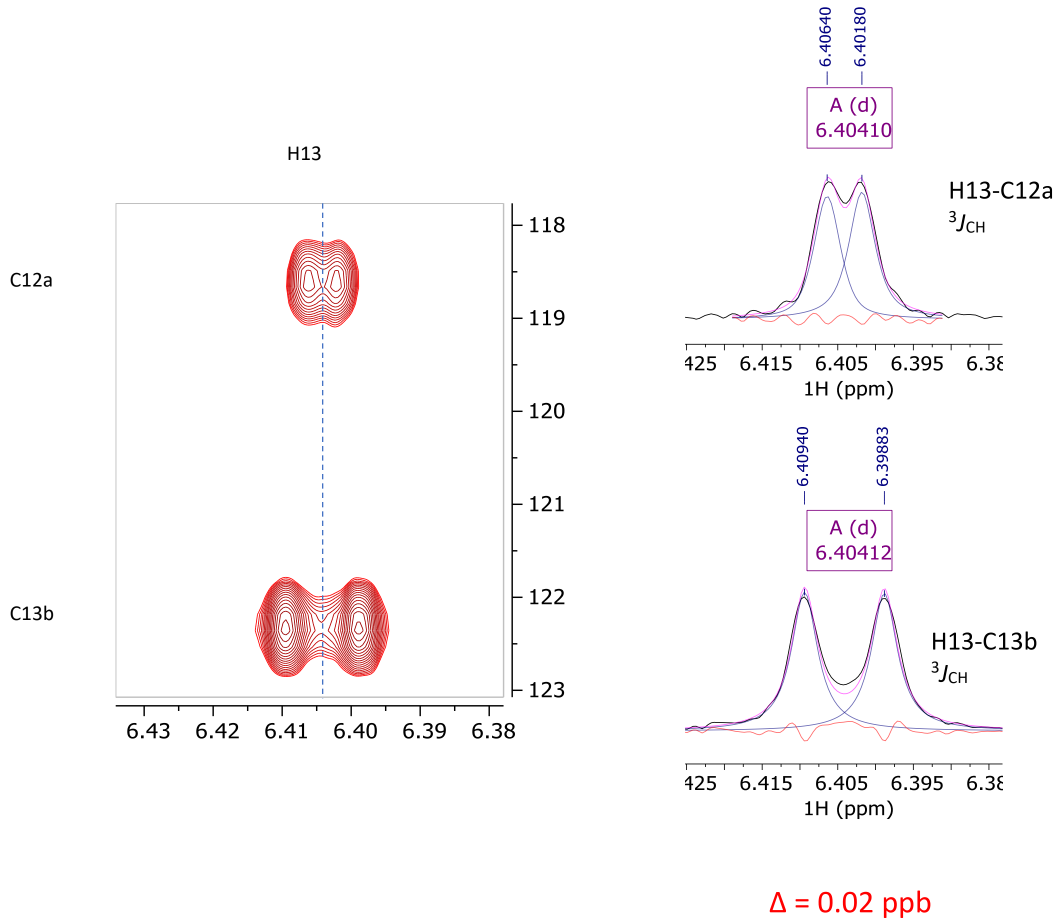

Supplementary Figure 20. ^{1}H spectrum of **13b** (600 MHz, CD_3OD, 298 K)

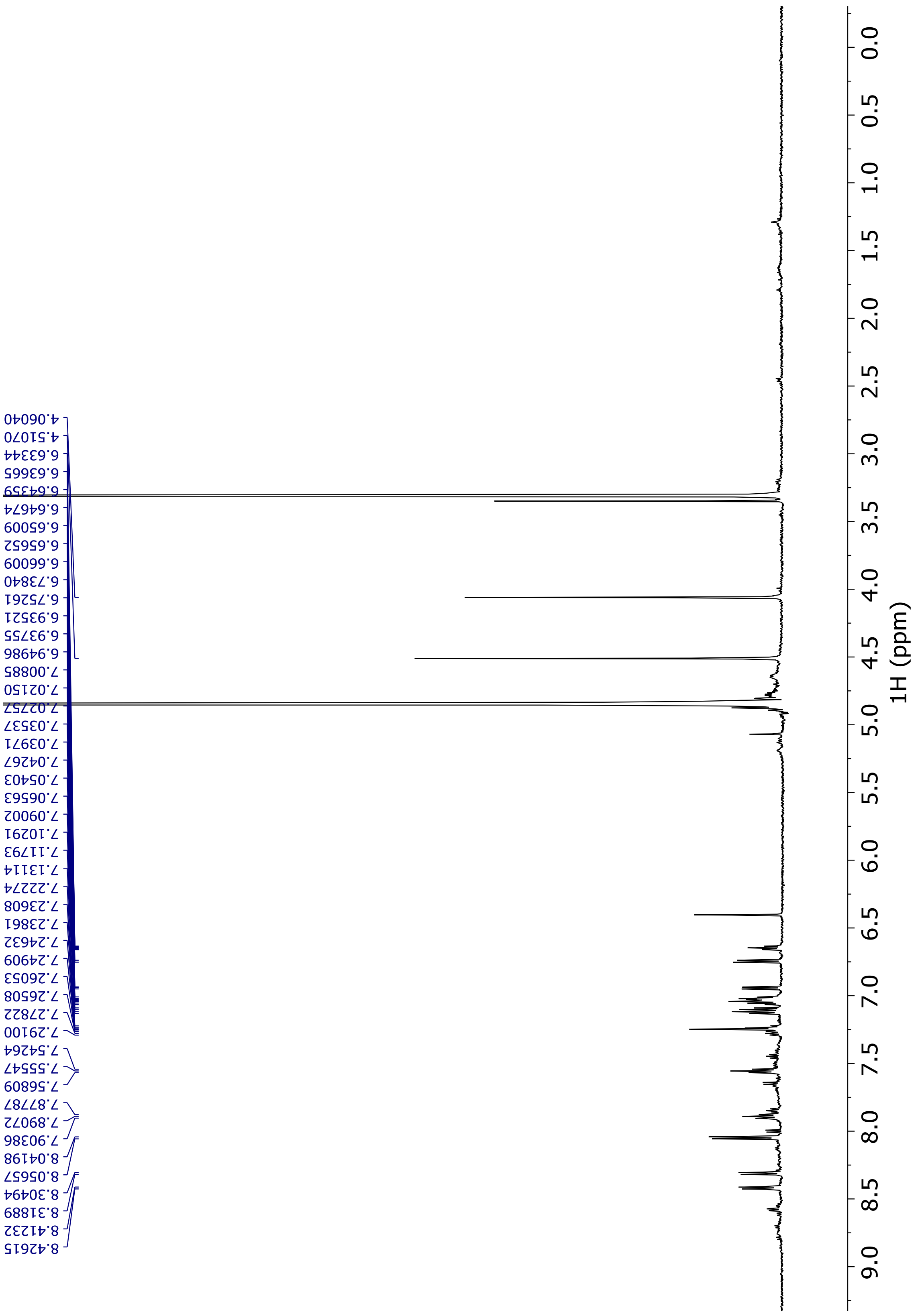

Supplementary Figure 21. ^{1}H-^{13}C i-HMBC spectrum of 13b (600 / 151 MHz, CD_3OD, 298 K)

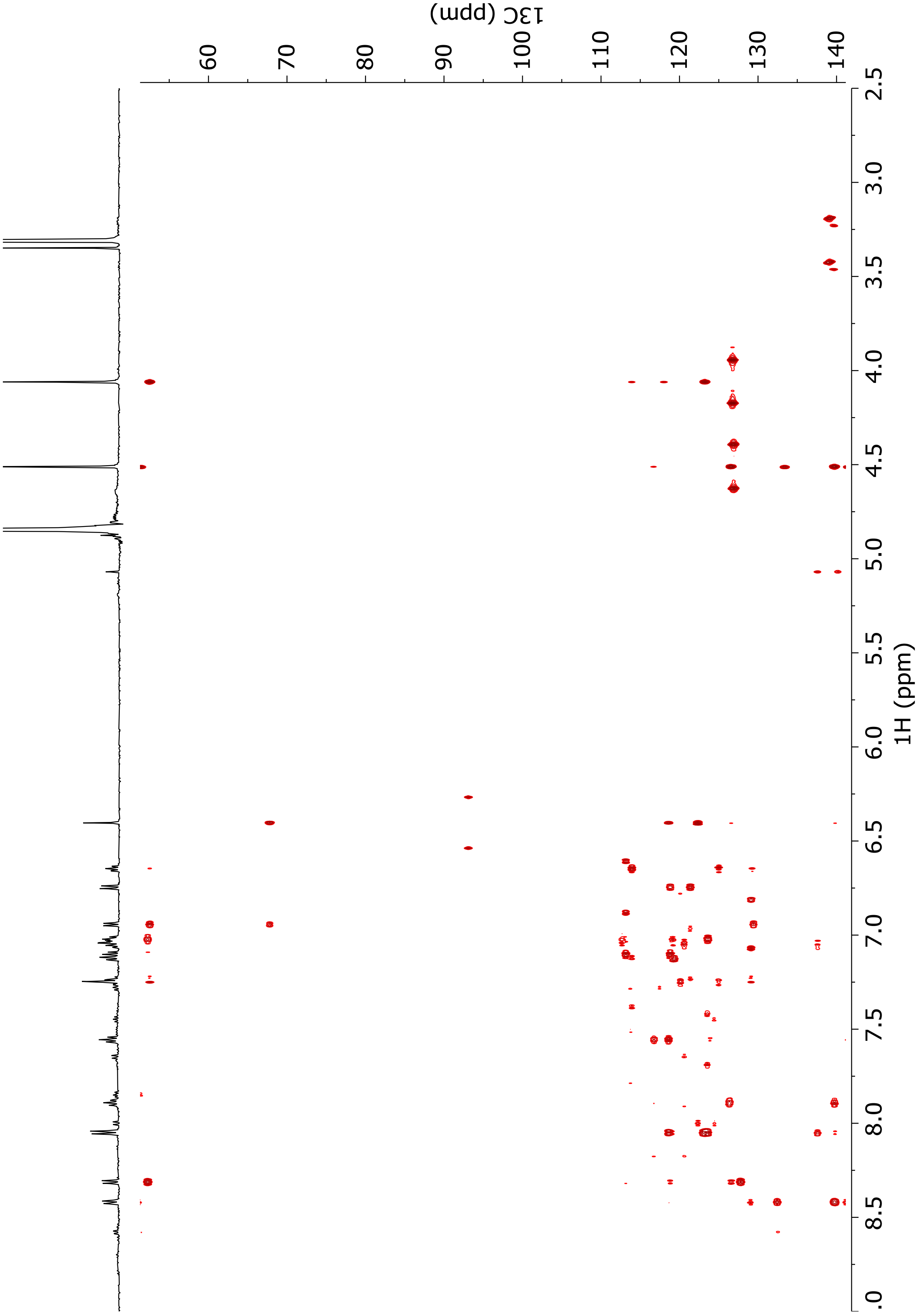

V. i-HMBC NMR Data and Spectra for Strychnine

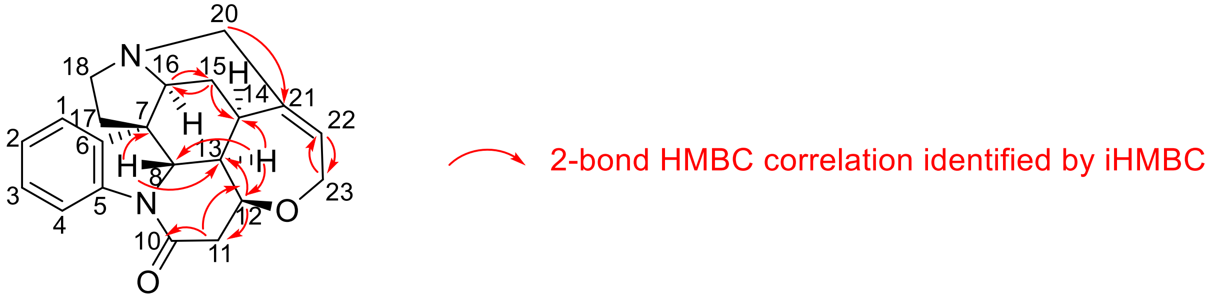

4

Strychnine (**4**):

^{1}H NMR (600 MHz, $CDCl_3$) δ 8.07 (ddd, *J* = 8.1, 1.1, 0.6 Hz, 1H), 7.24 (ddd, *J* = 8.0, 7.3, 1.4 Hz, 1H), 7.15 (dd, *J* = 7.4, 1.3 Hz, 1H), 7.08 (td, *J* = 7.5, 1.1 Hz, 1H), 5.94 – 5.85 (m, 1H), 4.27 (dt, *J* = 8.5, 3.3 Hz, 1H), 4.13 (dd, *J* = 13.8, 6.9 Hz, 1H), 4.05 (dddd, *J* = 13.8, 6.2, 1.7, 0.7 Hz, 1H), 3.95 (dd, *J* = 4.2, 2.2 Hz, 1H), 3.84 (d, *J* = 10.5 Hz, 1H), 3.70 (dq, *J* = 14.8, 1.6 Hz, 1H), 3.25 – 3.17 (m, 1H), 3.15 – 3.08 (m, 2H), 2.86 (td, *J* = 10.2, 8.3 Hz, 1H), 2.73 (d, *J* = 14.7 Hz, 1H), 2.65 (dd, *J* = 17.4, 3.3 Hz, 1H), 2.34 (dt, *J* = 14.4, 4.4 Hz, 1H), 1.92 – 1.84 (m, 2H), 1.44 (dt, *J* = 14.4, 2.1 Hz, 1H), 1.26 (dt, *J* = 10.5, 3.2 Hz, 1H).

^{13}C NMR (151 MHz, $CDCl_3$) δ 169.4, 142.3, 140.3, 132.7, 128.7, 127.7, 124.3, 122.4, 116.3, 77.7, 64.7, 60.3, 60.2, 52.8, 52.0, 50.4, 48.3, 42.9, 42.6, 31.7, 26.9.

Supplementary Table 3. Measured isotope shift differences for **4**, in ppb.

	H22	H12	H23a	H23b	H16	H8	H20a	H11a	H20b	H11b	H15a	H15b	H13
C15					-0.47				0				-0.13
C14	0	0			-0.27		-0.03		-0.07		-0.41	-0.36	-0.41
C11		-0.58											-0.13
C17						-0.22							
C13		-0.58	0	0		-0.45		0		-0.14	-0.10	-0.16	
C18							-0.24		-0.24				
C7		-0.01				-0.44					-0.12	-0.02	0
C20	-0.13		-0.03										
C8		-0.07			0								-0.51
C16						-0.19			-0.25		-0.51	-0.53	
C23	-0.60	-0.27					0						
C12			-0.13	-0.18		-0.04		-0.46		-0.58	-0.03		-0.37
C1						0							
C22			-0.49	-0.35			-0.15		-0.27				
C6					-0.11	-0.14							
C21			-0.20				-0.40		-0.41		0	0	-0.06
C5						-0.12		-0.03		0			
C10		-0.06						-0.47		-0.57			

Supplementary Figure 22. Selected i-HMBC slices of **4** showing chemical shifts and isotope shift measurements. (600 MHz, $CDCl_3$, 298 K)

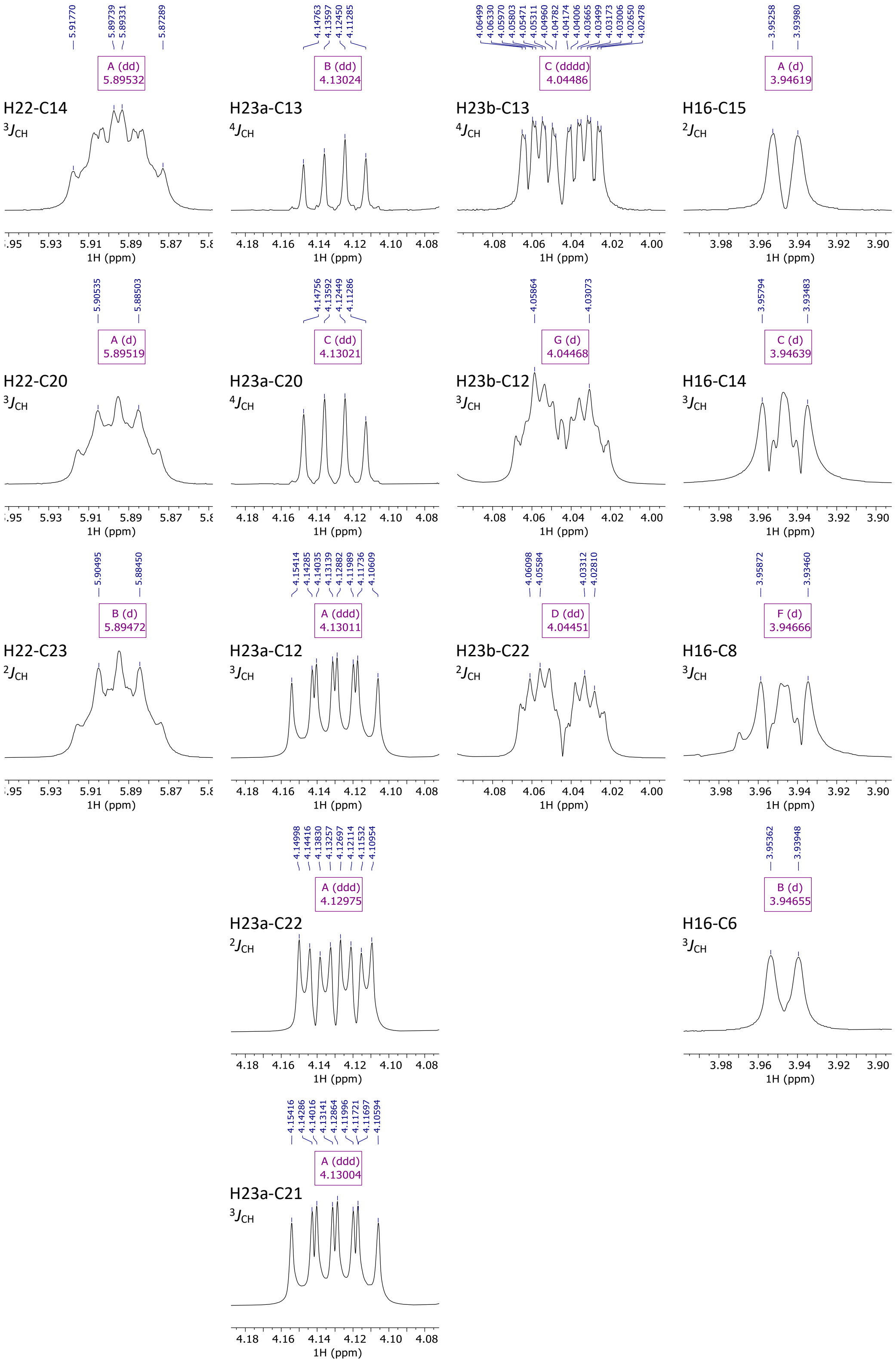

Supplementary Figure 22. (continued)

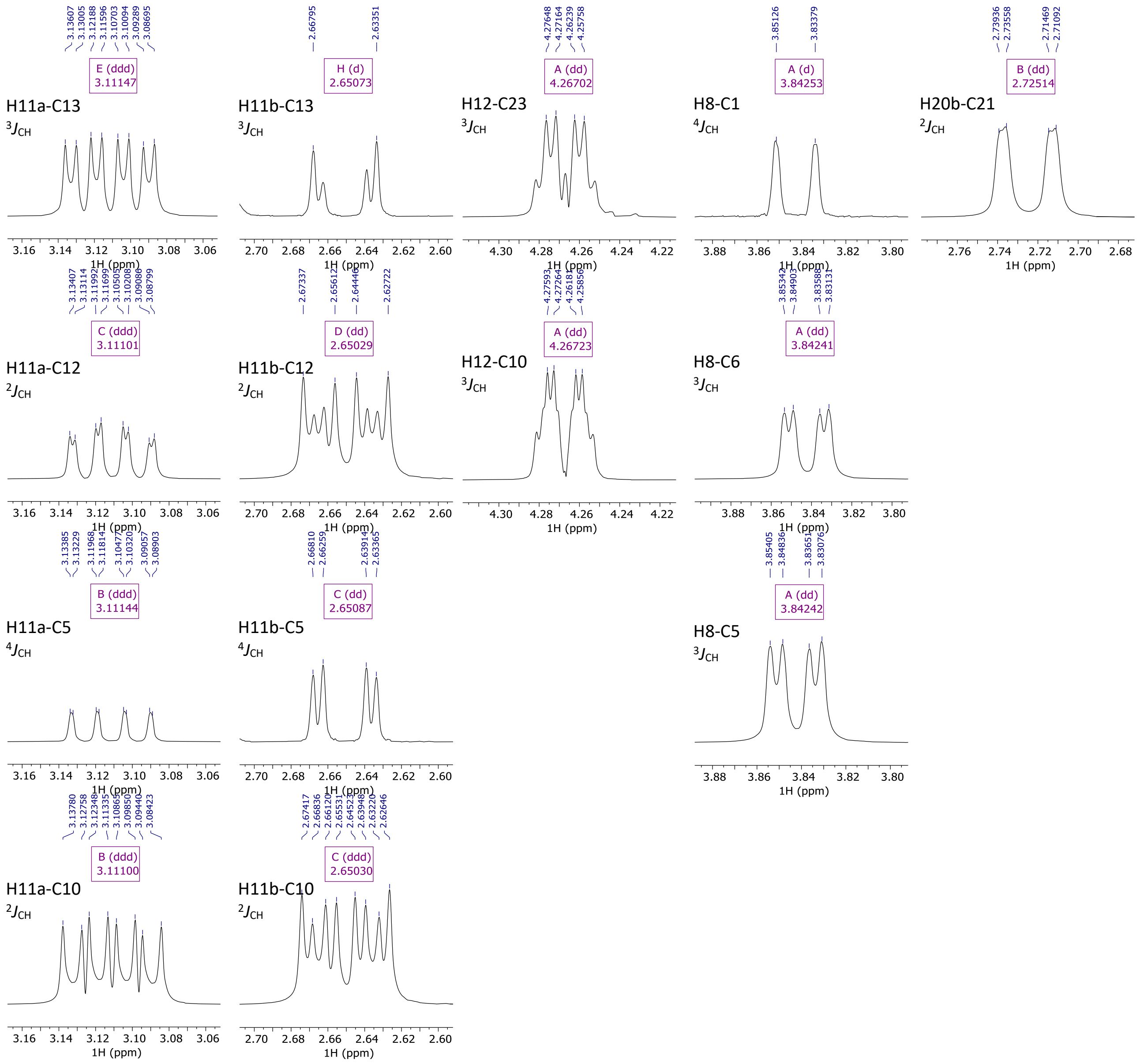

Supplementary Figure 22. (continued)

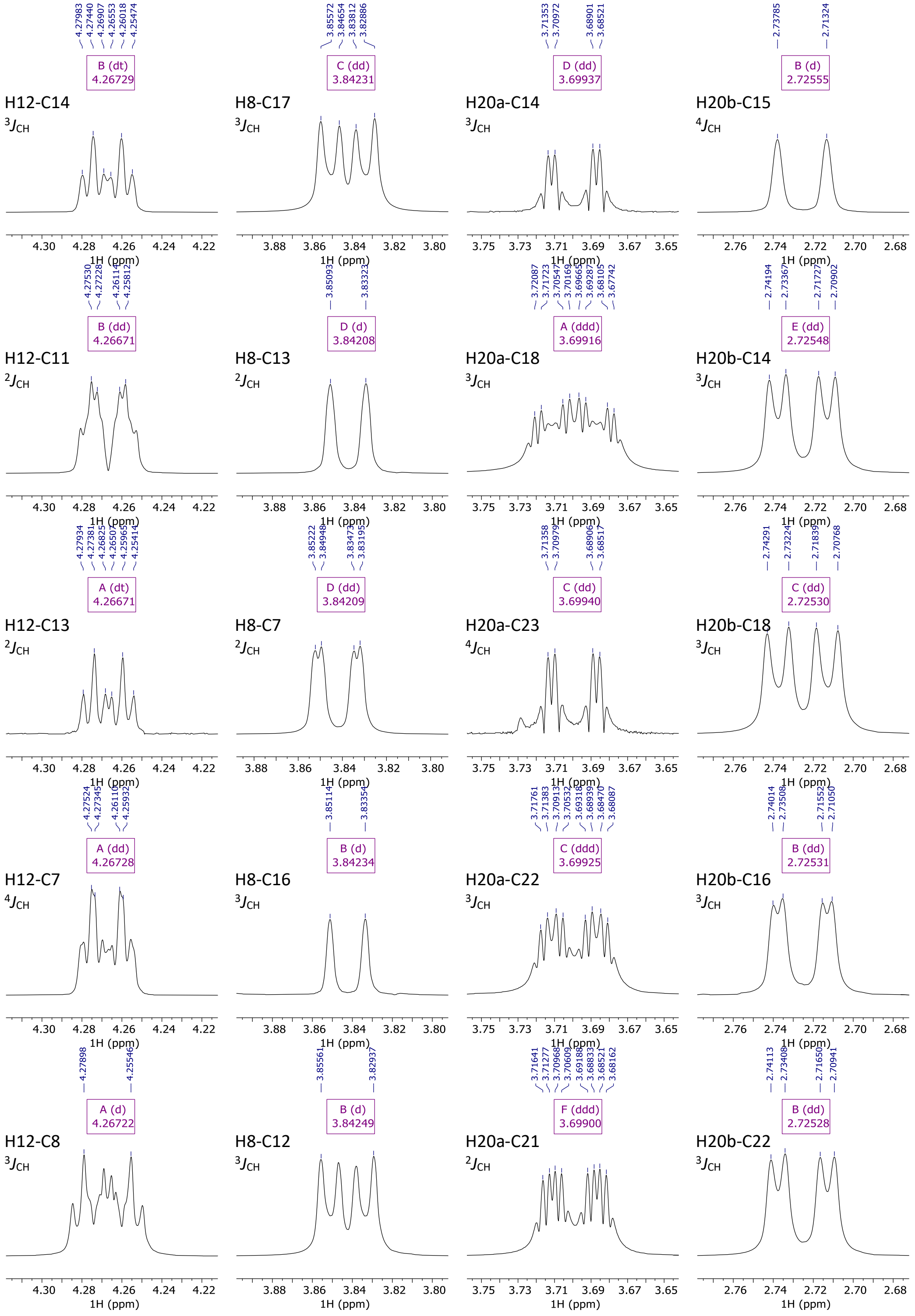

Supplementary Figure 22. (continued)

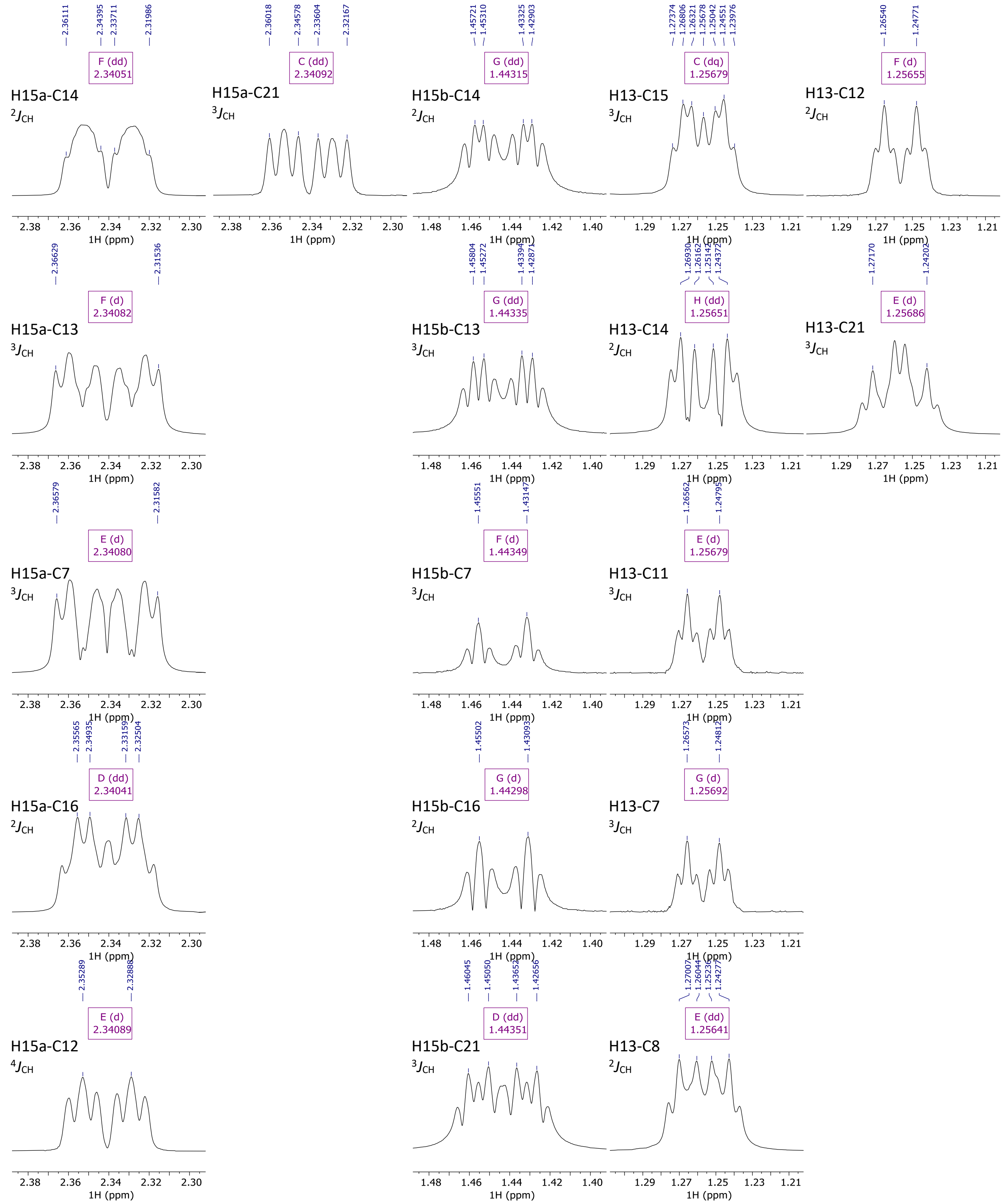

VI. i-HMBC NMR Data for Linalool, MTBE, Hydrochlorothiazide, Caffeine, Sulfamethoxazole, Prednisone, and Trimethoprim

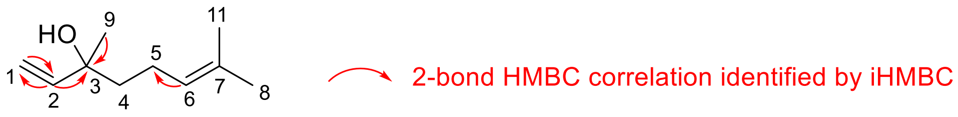

linalool (2)

Supplementary Table 4. Measured isotope shift differences for **2**, in ppb.

	H9	H2	H1'	H6	H1''
C11				-0.13	
C5	0			-0.29	
C8				0	
C9		-0.05	-0.07		-0.03
C4	-0.10	0	0	-0.07	0
C3	-0.61	-0.50	-0.06		-0.29
C1	-0.02	-1.44			
C2	-0.10		-1.52		-1.61

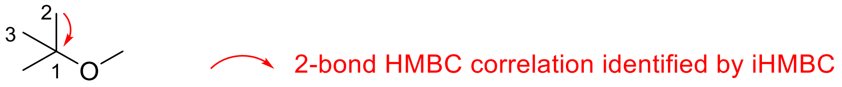

MTBE (3)

Supplementary Table 5. Measured isotope shift differences for **3**, in ppb.

	H2
C1	-0.45
C3	0

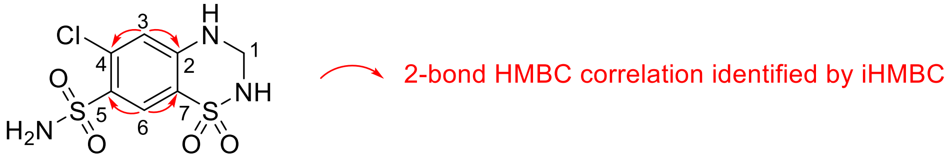

Hydrochlorothiazide (**5**)

Supplementary Table 6. Measured isotope shift differences for **5**, in ppb.

	H1	H3	H6
C2	0	-1.15	-0.11
C3	-0.19		-0.11
C4		-0.87	0
C5	-0.06	0	-0.77
C6		-0.07	
C7		-0.12	-0.94

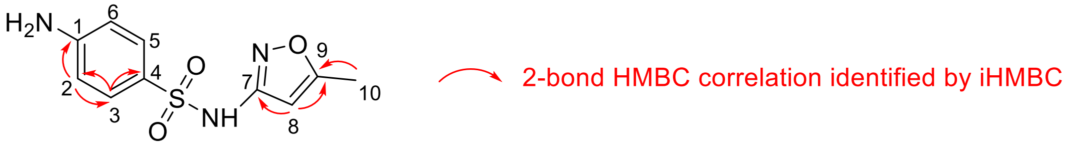

Sulfamethoxazole (**6**)

Supplementary Table 7. Measured isotope shift differences for **6**, in ppb.

	H2, H6	H3, H5	H8	H10
C1	-1.08	-0.14		
C2, C6	0	-0.54		
C3, C5	-0.75	0		
C4	-0.30	-0.70		
C9			-1.08	-0.55
C8				-0.05
C7			-0.66	0
C10			0	

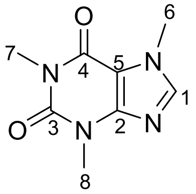

Caffeine (**7**)

Supplementary Table 8. Measured isotope shift differences for **7**, in ppb.

	H1	H6	H7	H8
C1		-0.05		
C2	-0.13		-0.04	-0.07
C3			-0.21	-0.24
C4	-0.06	0	-0.13	
C5	0	-0.04	0	0
C6	-0.01			

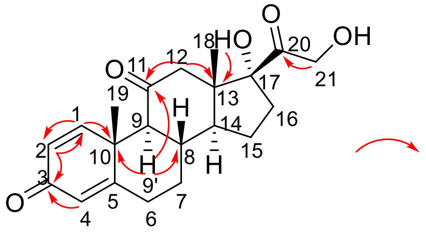

Prednisone (**8**)

Supplementary Table 9. Measured isotope shift differences for **8**, in ppb.

	H1	H2	H4	H9'	H12'	H12''	H18	H19	H21'	H21''
C1		-1.53						-0.24		
C2	-1.36							0		
C3	-0.14	-0.74	-0.88							
C4	0									
C5	-0.08	-0.11		-0.14				-0.13		
C6	-0.04		0					-0.25		
C7										
C8		0		-0.54	-0.25		0	-0.11		
C9	-0.13	-0.07	-0.04		-0.27			-0.08		
C10	-0.63	-0.48	-0.16	-0.75				-0.77		
C11				-0.58	-1.08	-0.98		-0.36		
C13					-0.44	-0.45	-0.57		0	0
C14				-0.03	-0.20	-0.07	-0.21			
C15					-0.08					
C16										
C17					-0.21	-0.21			-0.08	-0.22
C18					-0.24	0				
C19	-0.05	-0.06	-0.16	0						
C20									-0.50	-0.44
C21					0					

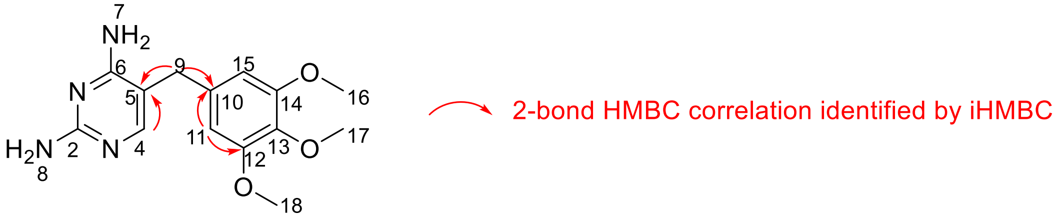

Trimethoprim (**9**)

Supplementary Table 10. Measured isotope shift differences for **9**, in ppb.

	H4	H7	H9	H11, H15	H16, H18
C2	-0.19	0			
C4			-0.19		
C5	-0.82	-0.16	-0.56	0	
C6	-0.10		-0.10		
C9	0			-0.18	
C10			-0.55	-1.02	
C13			0	-0.19	
C11, C15			-0.21	-0.36	0
C12, C14			-0.09	-0.57	-0.18

VII. i-HMBC and i-D-HMBC Pulse Sequences

Typically, the HMBC pulse sequence contains unbalanced gradient pulses, used to select desired coherence transfer pathways (CTPs). When the net gradient is not zero (i.e., unbalanced), the nuclei are never fully refocused, which includes the lock channel. Therefore, during the FID acquisition period, the lock system drifts in frequency. This causes signal lineshape distortions. By turning the lock off during acquisition, and relocking during the relaxation delay, D1, the correction by this drift is ignored, producing less distorted signal lineshapes. Also importantly, one needs to be sure that there is no residual current being induced in the gradient coil, which would distort the FID as extra inhomogeneity. Both requirements are made here with a simple arrangement of the commands BLKGRAMP (i.e., set coil current to zero, without locking the system) and LOCKH_ON/OFF (i.e., turn the lock hold on or off, which corrects the drift for the lock or not) in the pulse sequence. In addition, decreasing the lock power by 10-15 dBm was also found to reduce the lineshape distortions.

Modified HMBC pulse sequence to improve line shape for i-HMBC

```
;hmbcetgpnd
;avance-version (12/01/11)
;HMBC
;2D H-1/X correlation via heteronuclear zero and double quantum
;   coherence
;phase sensitive using Echo/Antiecho gradient selection
;no decoupling during acquisition
;
;modified from original hmbcetgpnd
;  updated 24/08/2022 RAHWAY
;
;  Guilherme Dal Poggetto and Xiao Wang
;  email: guilherme.dal.poggetto@merck.com and xiao.wang1@merck.com
;  MRL - Rahway, NJ
;
;D.O. Cicero, G. Barbato & R. Bazzo, J. Magn. Reson. 148,
;   209-213 (2001)
;
;$CLASS=HighRes
;$DIM=2D
;$TYPE=
;$SUBTYPE=
;$COMMENT=
```

```
#include <Avance.incl>
#include <Grad.incl>
#include <Delay.incl>

"cnst30=(1-sfo2/sfo1)/(1+sfo2/sfo1)"

define list<gradient> EA1 = { 1.000 -cnst30}
define list<gradient> EA2 = { -cnst30 1.000}

"p2=p1*2"
"d6=1s/(cnst13*2)"
"d0=3u"
"d11=30m"
"in0=inf1/2"
"DELTA1=p2+d0*2"

1 ze
2 d11
  50u LOCKH_OFF
  d1
  50u LOCKH_ON
  d11 pl1:f1
  50u UNBLKGRAMP
3 p1 ph1
  d6
  (p3 ph3):f2
  d0
  p2 ph2
  d0
  p16:gp1*EA1
  d16
  (p24:sp7 ph4):f2
  DELTA1
  p16:gp1*EA2
  d16 pl2:f2
  (p3 ph4):f2
  4u BLKGRAMP
  go=2 ph31
  d11 mc #0 to 2
     F1EA(calgrad(EA1) & calgrad(EA2), caldel(d0, +in0) & calph(ph3, +180) & calph(ph31, +180))
  20u LOCKH_OFF
exit
```

```
ph1=0
ph2=0 0 2 2
ph3=0 2
ph4=0 0 0 0 2 2 2 2
ph31=0 2 0 2 2 0 2 0

;pl1 : f1 channel - power level for pulse (default)
;pl2 : f2 channel - power level for pulse (default)
;sp7: f2 channel - shaped pulse (180degree refocussing)
;spnam7: Crp60comp.4
;p1 : f1 channel -  90 degree high power pulse
;p2 : f1 channel - 180 degree high power pulse
;p3 : f2 channel -  90 degree high power pulse
;p16: homospoil/gradient pulse                    [1 msec]
;p24: f2 channel - 180 degree shaped pulse for refocussing
;     = 2msec for Crp60comp.4
;d0 : incremented delay (2D)                      [3 usec]
;d1 : relaxation delay; 1-5 * T1
;d6 : delay for evolution of long range couplings (1/2Jlr)
;d16: delay for homospoil/gradient recovery
;cnst13: = J(XH) long range
;inf1: 1/SW(X) = 2 * DW(X)
;in0: 1/(2 * SW(X)) = DW(X)
;nd0: 2
;ns: 2 * n
;ds: 16
;td1: number of experiments
;FnMODE: echo-antiecho

;use gradient ratio:    gp 1
;                       80
;for z-only gradients:
;gpz1: 80%

;use gradient files:
;gpnam1: SMSQ10.100

;Processing
;PH_mod(F1): pk (or no)
;use xfb and xf2m

;$Id:$
```

Modified D-HMBC pulse sequence to improve line shape for D-i-HMBC with mild adiabatic decoupling

Supplementary Figure 23. D-i-HMBC pulse sequence

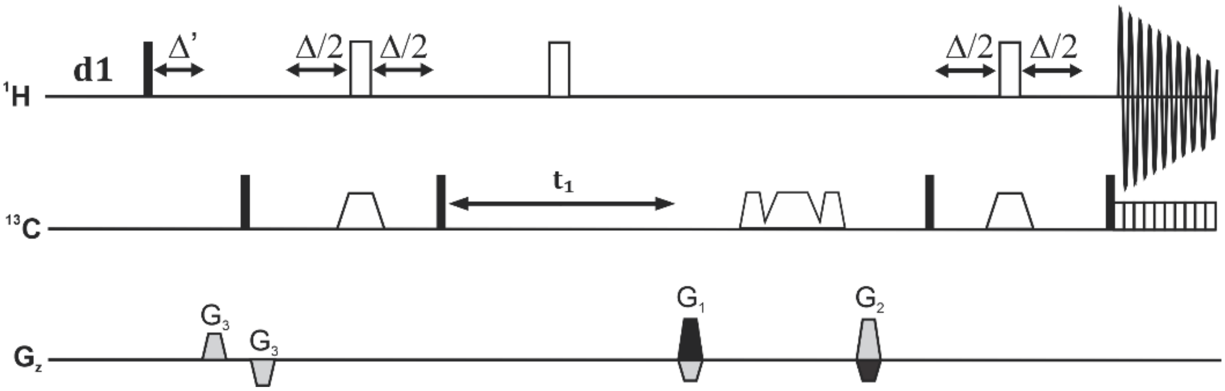

<u>Note</u>: The adiabatic decoupling pulse was achieved using wavemaker with cawurst-d-40: 200 ppm decoupling bandwidth (CNST51) and 20 ms decoupling pulse length (CNST52).

```
;dhmbcetgp_adia
; updated 24/08/2022 RAHWAY
;
;   Guilherme Dal Poggetto and Xiao Wang
;   email: guilherme.dal.poggetto@merck.com and xiao.wang1@merck.com
;   MRL - Rahway, NJ
;
;CLIP-D-HMBC
;2D H-1/X correlation via heteronuclear zero and double quantum
;   coherence
;with low pass J-filter
;phase sensitive using Echo/Antiecho gradient selection
;with adiabatic decoupling (wvm) during acquisition
;
;K. Furihata & H. Seto. Tetrahedron. Lett. 36, 2817-2820 (1995)
;D.O. Cicero, G. Barbato & R. Bazzo, J. Magn. Reson. 148,
;   209-213 (2001)
;P. Bigler & J. Furrer. Magn. Reson. Chem. 57, 129-143 (2019)
;
;$CLASS=HighRes
;$DIM=2D
;$TYPE=
;$SUBTYPE=
;$COMMENT=
```

```
#include <Avance.incl>
#include <Grad.incl>
#include <Delay.incl>

"cnst30=(1-sfo2/sfo1)/(1+sfo2/sfo1)"

define list<gradient> EA1 = { 1.000 -cnst30}
define list<gradient> EA2 = { -cnst30 1.000}

"p2=p1*2"
"d6=1s/(cnst6*2)"
"d4=1s/(cnst13*4)"
"d11=30m"
"d11=30m+1s/cnst51-1s/cnst51"
"d11=30m+1s/cnst52-1s/cnst52"

"d0=3u"

"in0=inf1/2"

"DELTA1=p2+d0*2"
"DELTA=d4-larger(p2,p14)/2"
"DELTA2=DELTA+d6/2"
"DELTA3=DELTA2-40u-p3"

1 ze
2 d11 do:f2
  50u LOCKH_OFF
  d1
  50u LOCKH_ON
  d11 pl1:f1 pl2:f2
  50u UNBLKGRAMP

3 p1 ph1
  d6

  p16:gp3
  (p3 ph2):f2
  p16:gp3*-1

  DELTA pl0:f2
  (center (p2 ph1) (p14:sp3 ph1):f2 )
  DELTA pl2:f2
```

```
  (p3 ph3):f2
  d0
  p2 ph4
  d0

  p16:gp1*EA2
  d16 pl0:f2
  (p24:sp7 ph5):f2
  DELTA1
  p16:gp1*EA1
  d16 pl2:f2
  (p3 ph5):f2

  DELTA2 pl0:f2
  (center (p2 ph1) (p14:sp3 ph1):f2 )
  DELTA3
  30u BLKGRAMP
  (p3 ph7):f2
  10u pl12:f2

  go=2 ph31 cpd2:f2
  d11 do:f2 mc #0 to 2
    F1EA(calgrad(EA1) & calgrad(EA2), caldel(d0, +in0) & calph(ph5, +180) & calph(ph31, +180))
  20u LOCKH_OFF
exit

ph1=0
ph2=0 0 2 2
ph3=0 2
ph4=0 0 0 0 2 2 2 2
ph5=0 0 0 0 0 0 0 0 2 2 2 2 2 2 2 2
ph7=0
ph31=0 2 0 2 0 2 0 2 2 0 2 0 2 0 2 0

;pl1 : f1 channel - power level for pulse (default)
;pl2 : f2 channel - power level for pulse (default)
;sp7: f2 channel - shaped pulse (180degree refocussing)
;spnam7: Crp60comp.4
;p1 : f1 channel -  90 degree high power pulse
;p2 : f1 channel - 180 degree high power pulse
;p3 : f2 channel -  90 degree high power pulse
;p16: homospoil/gradient pulse                    [1 msec]
;p24: f2 channel - 180 degree shaped pulse for refocussing
;     = 2msec for Crp60comp.4
```

```
;d0 : incremented delay (2D)                    [3 usec]
;d1 : relaxation delay; 1-5 * T1
;d6 : delay for low pass J-filter
;d16: delay for homospoil/gradient recovery
;cnst6: = 1J(XH) one bond coupling
;cnst13: = J(XH) long range coupling
;inf1: 1/SW(X) = 2 * DW(X)
;in0: 1/(2 * SW(X)) = DW(X)
;nd0: 2
;ns: 2 * n
;ds: 16
;td1: number of experiments
;FnMODE: echo-antiecho
;sp15(pcpd2,pl12):wvm:wuCdec:f2 cawurst_d-40(cnst51 ppm, cnst52 ms; Q=2)
;cnst51: decoupling bandwidth (ppm)
;cnst52: decoupling pulse length (=1/5J, ms)

;use gradient ratio:    gp 1 : gp 3
;                       80 : 54.4
;for z-only gradients:
;gpz1: 80%
;gpz3: 54.4%
;use gradient files:
;gpnam1: SMSQ10.100
;gpnam3: SMSQ10.100

;Processing
;PH_mod(F1): pk (or no)
;use xfb and xf2m

;$Id:$
```

VIII. S/N comparison of HMBC, i-HMBC and 1,1-ADEQUATE Pulse Sequences

The signal-to-noise (S/N) ratio of the following four H-C correlations of strychnine in 600 µL $CDCl_3$ were measured with identical recycling time (2.3 s) using a 600 MHz Bruker Neo NMR spectrometer equipped with a 5 mm QCI cryoprobe. These were used to compare different NMR experiments with the same overall experiment time.

Supplementary Figure 24. H-C correlations of strychnine used for S/N comparison.

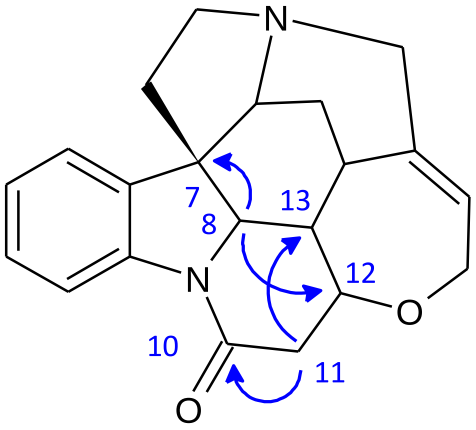

For HMBC, Bruker pulse sequence hmbcetgpnd was used, with D1 = 2.1 s, AQ = 0.2 s, J_{opt} = 8 Hz.

For i-HMBC, the pulse sequence in section VII was used, with D1 = 0.3 s, AQ = 2 s, J_{opt} = 8 Hz.

For 1,1-ADEQUATE, Bruker pulse sequence adeq11etgprdsp was used, with D1 = 2.1 s, AQ = 0.2 s, J_{CH} = 145 Hz, J_{CC} = 45 Hz.

For 10 mg and 1 mg strychnine, 256 F1 increments were used. For 0.2 mg strychnine, 128 F1 increments were used. During processing, 4x zero-filling was applied to ^1H dimension for all experiments.

Supplementary Table 11. S/N comparison for HMBC, i-HMBC and 1,1-ADEQUATE experiments.

10 mg Strychnine	Measured J_{CH} (Hz)[1]	S/N				
		HMBC	i-HMBC	i-HMBC (50% NUS)	1,1-ADEQUATE	1,1-ADEQUATE
Expt. Time		20 min	21 min	11 min	20 min	161 min
H11a-C10	6.4	682	299	362	N.D.	4.8
H11a-C13	3.7	383	202	220	-	-
H8-C7	2.2	349	180	224	4.7	14
H8-C12	5.6	879	312	368	-	-

N.D. = not detectable

i-HMBC experiments are a few minutes longer than HMBC and 1,1-ADQUATE experiments using same AQ+D1 and number of scans due to additional delays of d11 = 30 ms in the pulse sequence before d1 (SI section VII).

1 mg Strychnine	Measured J_{CH} (Hz)	S/N				
		HMBC	i-HMBC	i-HMBC (50% NUS)	1,1-ADEQUATE	1,1-ADEQUATE
Expt. Time		2.7 h	2.8 h	1.4 h	2.7 h	21 h
H11a-C10	6.4	159	67	76	N.D.	N.D.
H11a-C13	3.7	114	47	55	-	-
H8-C7	2.2	78	43	49	N.D.	4.1
H8-C12	5.6	196	71	78	-	-

N.D. = not detectable

0.2 mg Strychnine	Measured J_{CH} (Hz)	S/N				
		HMBC	i-HMBC	i-HMBC (50% NUS)	1,1-ADEQUATE	1,1-ADEQUATE
Expt. Time		43 h	44 h	22 h		
H11a-C10	6.4	121	52	50	-	-
H11a-C13	3.7	95	32	33	-	-
H8-C7	2.2	65	35	31	-	-
H8-C12	5.6	134	56	59	-	-

1,1-ADEQUATE was not measured for 0.2mg strychnine due to low sensitivity.

IX. Comparison of measurement accuracy of signals with different S/N using peak picking versus line fitting, and effect of NUS

The i-HMBC of a sample of EtOAc in 600 μL $CDCl_3$ was measured in a 600 MHz Bruker Avance III HD spectrometer equipped with a 5 mm TCI cryoprobe, with D1 = 0.5 s, AQ = 2 s, SW = 10 ppm, points in ^{1}H dimension = 24036 (or 12018 complex points), FIDRES = 0.5 Hz, F1 increment = 64. Different number of scans (NS) were used to achieve different S/N. Each spectrum was then zero-filled (ZF) to a total of either 16 k complex points (digital resolution 0.37 Hz or 0.61 ppb), 32k complex points (digital resolution 0.18 Hz or 0.31 ppb), or 64 k complex points (digital resolution 0.09 Hz or 0.15 ppb) in ^{1}H dimension during processing. Each experiment was repeated three times.

For peak picking, GSD method in Mnova (v14.2.1) was used, with refinement level 4 (20 fitting cycles), and quantitative GSD with 10 improvement cycles.

For line fitting, Generalized Lorentzian was used with 5000 maximum number of coarse iterations, 5000 maximum number of fine iterations, and local minima filter set to 5. The fitting was iterated until a stable fitting residual was reached.

The reference value of $^{2\text{-}3}\Delta^{1}H(^{13/12}C)$ was measured to be -0.25 – -0.26 ppb by isotope labeling and selective ^{13}C decoupling experiments (Figure 1).

Results of $^{2\text{-}3}\Delta^{1}H(^{13/12}C)$ measurements via i-HMBC are shown below in Table S12.

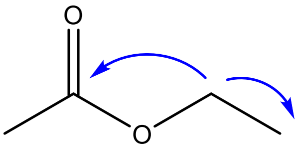

Supplementary Table 12. Measurement accuracy of $^{2\text{-}3}\Delta^1H(^{13/12}C)$ in ethyl acetate via i-HMBC depending on spectral resolution and S/N.

S/N	$^{2\text{-}3}\Delta^1H(^{13/12}C)$, in ppb, obtained by line fitting method			
	4x ZF, 0.15 ppb	2x ZF, 0.31 ppb	1x ZF, 0.61 ppb	4x ZF, 0.15 ppb, 50% NUS
17	-0.26 ± 0.11	-0.23 ± 0.08	-0.26 ± 0.11	-0.16 ± 0.09
35	-0.28 ± 0.03	-0.27 ± 0.05	-0.28 ± 0.03	-0.31 ± 0.04
70	-0.28 ± 0.01	-0.29 ± 0.02	-0.28 ± 0.01	-0.34 ± 0.02

S/N	$^{2\text{-}3}\Delta^1H(^{13/12}C)$, in ppb, obtained by peak picking method			
	4x ZF, 0.15 ppb	2x ZF, 0.31 ppb	1x ZF, 0.61 ppb	4x ZF, 0.15 ppb, 50% NUS
17	-0.26 ± 0.07	-0.29 ± 0.11	-0.32 ± 0.04	-0.19 ± 0.01
35	-0.27 ± 0.04	-0.20 ± 0.07	-0.28 ± 0.05	-0.23 ± 0.09
70	-0.28 ± 0.02	-0.24 ± 0.06	-0.29 ± 0.05	-0.28 ± 0.03

Both peak picking and line fitting methods yield comparable and accurate $^{2\text{-}3}\Delta^1H(^{13/12}C)$ values when S/N ≥ 35 without NUS.

50% NUS would shorten the experiment time by half, but it could yield larger errors especially for low S/N signals. Although based on our experience, an error within 0.05 ppb usually is tolerable when used to differentiate two-bond HMBC from longer-range HMBC. Caution should be used when trying to apply NUS to i-HMBC, and for low S/N samples NUS is not recommended.

X. Nomenclature of isotope shift

Isotope effects are defined as $^{n}\Delta X(H/L) = \delta X(L)-\delta X(H)$, where n denotes the number of bonds between the nucleus investigated; $\delta X(H)$ is the nuclear shielding of nucleus X substituted with the heavier isotope; $\delta X(H)$ is the nuclear shielding of nucleus X substituted with the lighter isotope.[2,3]

Thus a two-bond ^{13}C isotope effect on ^{1}H chemical shift is denoted as $^{2}\Delta^{1}H(^{13}/^{12}C)$, and a three-bond isotope shift is denoted as $^{3}\Delta^{1}H(^{13}/^{12}C)$. Correspondingly, the difference between two- and three-bond isotope shift, the relative isotope shift discussed in this work, is denoted as $^{2\text{-}3}\Delta^{1}H(^{13}/^{12}C)$.

Supplementary References

1. Koos, MR, Navarro-Vázquez, A, Anklin, C, Gil, RR. Computer-Assisted 3D Structure Elucidation (CASE-3D): The Structural Value of $^{2}J_{CH}$ in Addition to $^{3}J_{CH}$ Coupling Constants. *Angew. Chem. Int. Ed.* **59**(10), 3938–3941 (2020).
2. Hansen, PE. Isotope Effects in Nuclear Shielding. *Prog. Nucl. Magn. Reson. Spectrosc*. **20**, 207–255 (1988).
3. Jameson, CJ. Isotope Effects on Chemical Shifts and Coupling Constants. *eMagRes*, 2007 John Wiley & Sons, Ltd. DOI: 10.1002/9780470034590.emrstm0251
